# Insights into the structure and function of the rate-limiting enzyme of chlorophyll degradation through analysis of a bacterial Mg-dechelatase homolog

**DOI:** 10.1016/j.csbj.2021.09.023

**Published:** 2021-09-23

**Authors:** Debayan Dey, Dipanjana Dhar, Helena Fortunato, Daichi Obata, Ayumi Tanaka, Ryouichi Tanaka, Soumalee Basu, Hisashi Ito

**Affiliations:** a Graduate School of Life Science, Hokkaido University, Sapporo 060-0810, Japan; b Graduate School of Science, Hokkaido University, Sapporo 060-0810, Japan; c Department of Natural History Sciences, Hokkaido University, Sapporo 060-0810, Japan; d Institute of Low Temperature Science, Hokkaido University, Sapporo 060-0819, Japan; e Department of Microbiology, University of Calcutta, Kolkata 700019, India

**Keywords:** Mg-dechelatase, Stay-Green, *Anaerolineae*, Chlorophyll degradation, Protein structure prediction, Molecular dynamics simulation

## Abstract

The Mg-dechelatase enzyme encoded by the *Stay-Green* (*SGR*) gene catalyzes Mg^2+^ dechelation from chlorophyll *a.* This reaction is the first committed step of chlorophyll degradation pathway in plants and is thus indispensable for the process of leaf senescence. There is no structural information available for this or its related enzymes. This study aims to provide insights into the structure and reaction mechanism of the enzyme through biochemical and computational analysis of an SGR homolog from the Chloroflexi *Anaerolineae* (AbSGR-h). Recombinant AbSGR-h with its intact sequence and those with mutations were overexpressed in *Escherichia coli* and their Mg-dechelatase activity were compared. Two aspartates – D34 and D62 were found to be essential for catalysis, while R26, Y28, T29 and D114 were responsible for structural maintenance. Gel filtration analysis of the recombinant AbSGR-h indicates that it forms a homo-oligomer. The three-dimensional structure of AbSGR-h was predicted by a deep learning-based method, which was evaluated by protein structure quality evaluation programs while structural stability of wild-type and mutant forms were investigated through molecular dynamics simulations. Furthermore, in concordance with the results of enzyme assay, molecular docking concluded the significance of D34 in ligand interaction. By combining biochemical analysis and computational prediction, this study unveils the detailed structural characteristics of the enzyme, including the probable pocket of interaction and the residues of structural and functional importance. It also serves as a basis for further studies on Mg-dechelatase such as elucidation of its reaction mechanism or inhibitor screening.

## Graphical abstract

**Figure:**
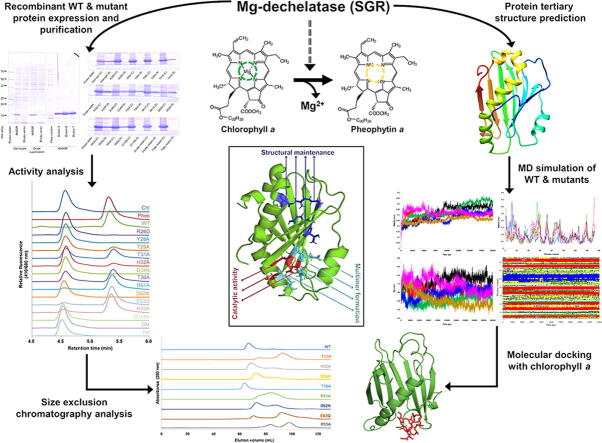

## 1 Introduction

Chlorophyll is a pigment that plays a crucial role in absorption, transmission and transformation of light energy during photosynthesis. Land plants and green algae have chlorophyll *a* and *b* as their photosynthetic pigments. Chlorophyll biosynthesis must be finely regulated for efficient photosynthetic performance during the formation of photosystems at the greening stage and also during adaptation to various environmental conditions [1]. Not only chlorophyll biosynthesis but also chlorophyll degradation needs to be regulated because the latter plays a crucial role in mobilizing resources from chloroplast to developing organs [2]. In addition, chlorophyll breakdown forms a key part of nitrogen recycling and is important in avoiding cellular photodamage. The major pathway and enzymes involved in chlorophyll degradation have been determined [3]. The first step of the degradation process is the extraction of magnesium (Mg) ion from chlorophyll *a* to form pheophytin *a* by Mg-dechelatase encoded by the *Stay-Green* (SGR) gene, which is also responsible for Mendel’s green-cotyledon peas [4]. Furthermore, this reaction is strictly regulated to prevent the formation of detrimental photoreactive chlorophyll intermediates, thus serving as the rate-limiting step of the chlorophyll breakdown pathway [5], [6]. The *Arabidopsis* SGR-less mutants showed substantial retardation of chlorophyll degradation during senescence [7], while overexpression of SGR resulted in the early promotion of chlorophyll degradation [8], [6].

**Fig. 1:**
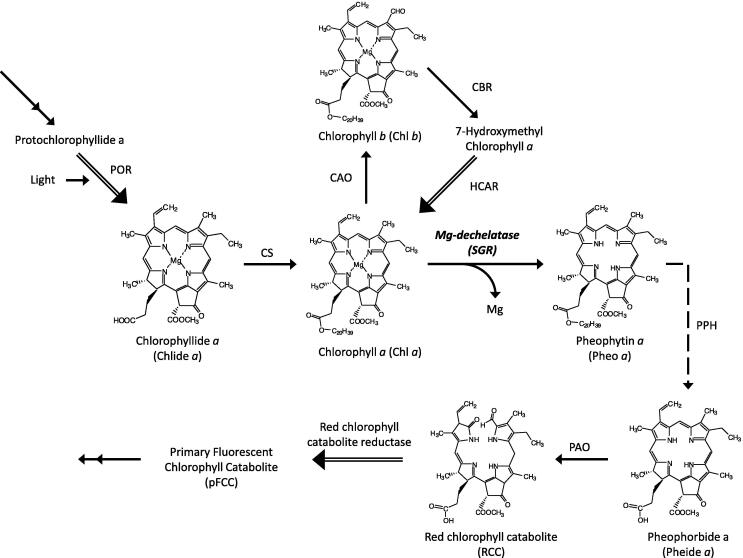
Chlorophyll degradation pathway in green plants. The enzymes that catalyze the reaction represented by double lined arrows have entry in the Protein Data Bank. Dash lined arrow indicates enzyme whose structure had been predicted using computational approaches. Structural information of enzymes involving reactions represented by normal arrows is awaited till when the work was done. CAO, chlorophyllide *a* oxygenase; CBR, chlorophyll *b* reductase; CS, chlorophyll synthase; HCAR, 7-hydroxymethyl chlorophyll *a* reductase; PPH, pheophytin pheophorbide hydrolase; POR, NADPH: protochlorophyllide oxidoreductase.

Chlorophyll biosynthesis shares the common biosynthetic pathway with other tetrapyrroles such as heme, siroheme and phycobilins [9]. Chlorophyll is structurally similar to heme with regard to the tetrapyrrole macrocycle ring but contains a central magnesium ion instead of iron. Crystal structures of enzymes involved in heme biosynthesis were extensively investigated [10]. In contrast, structural aspects of the enzymes related to the chlorophyll metabolic pathway remained unknown until recently, when the structures of Mg-chelatase and light dependent protochlorophyllide oxidoreductase were reported [11], [12]. Both of these enzymes catalyze the regulatory steps of chlorophyll biosynthesis. For chlorophyll breakdown, chlorophyll *b* must be converted to chlorophyll *a* by two successive reduction reactions because chlorophyll *b* derivatives are not catalyzed in the later steps of the chlorophyll degradation pathway [13]. In other words, the removal of Mg^2+^ from chlorophyll *b* leads to the formation of toxic pheophorbide *b* molecule which cannot be converted into another metabolite and induces a cell-death phenotype [14]. In the first step of chlorophyll *b* conversion, the enzyme chlorophyll *b* reductase (CBR) reduces the formyl group of chlorophyll *b* to produce 7-hydroxymethyl chlorophyll *a* [15]. In the final step, chlorophyll *a* is formed by the enzyme 7-hydroxymethyl chlorophyll *a* reductase (HCAR), the structure of which resembles an archaeal F_420_-reducing [NiFe] hydrogenase [16]. Furthermore, chlorophyll *a* is turned into a primary fluorescent Chl catabolite (pFCC) by four continuous steps (Fig. 1). Among the enzymes catalyzing these four steps of chlorophyll degradation, the crystal structure of only red chlorophyll catabolite reductase has been reported [17] while catalytic and structural properties of pheophytinase was investigated *in silico* [18]. However, the structure of Mg-dechelatase, catalyzing the committed step of the chlorophyll degradation pathway, is still unavailable.

Among eukaryotic photosynthesizing organisms, SGR is present only in green plants and Glaucophyta. On the other hand, SGR homologs are widely distributed in non-photosynthetic bacteria and Archaea [19]. Despite the high sequence similarity between SGR and its homologs, their catalytic activity and substrate specificity vary considerably among species. According to Obata et al. [19], a few bacterial SGR homologs, which are phylogenetically close to eukaryotic SGRs, show high Mg-dechelating activity and broad substrate specificity, suggesting the horizontal transfer of bacterial SGR homolog to photosynthetic eukaryotes.

SGR not only catalyzes the committed step of chlorophyll degradation, but also removes a metal ion from an organic compound in a chemically rare event. Therefore, extensive study on SGR will provide insights into a new type of enzymatic reaction mechanism. Besides, the substrate condition of SGR is different from conventional enzymes as it remains bound to the surface of chlorophyll-protein complexes instead of commonly free and small molecules that can fit into the active site of an enzyme. Thus, the active site of SGR needs to be exposed to the surface of the enzyme.

Preparation and purification of recombinant SGR with high yields is essential for performing detailed biochemical analyses of the enzyme. However, expression of recombinant SGR in large amounts without compromising the activity remains challenging and invariably results in insolubility. Even if it becomes soluble, the expressed enzyme precipitates on increasing the concentration making purification and further enzymatic analyses difficult. Though SGR from *Chlamydomonas reinhardtii* had been expressed in *E. coli* [20], plant SGRs were not expressed before. The first recombinant plant SGR was obtained using a cell-free protein expression system [6]. Mutant *Arabidopsis* SGR-Like and rice SGR were also obtained using the same *in vitro* expression system [21], [22]. Later, using an expression vector with a tag containing an unstructured and hyper-acidic module [23], *Arabidopsis* SGR and bacterial SGR homolog expression in *E. coli* were reported [19]. However, the expression levels for both the proteins did not increase substantially. In this study, we report the first successful overexpression of a soluble and highly active SGR homolog from *Anaerolineae* bacterium SM23_63 in *E. coli* using its general expression vector.

The evolutionary aspects of SGR coupled with its functional importance in plant senescence make it a molecule of utmost significance, the structural information of which emerges out to be absolutely vital. We therefore elucidated the structural characteristics of the protein through biochemical experiments and subsequently justified our observations using computational approaches.

## 2 Materials and methods

### 2.1 Multiple sequence alignment and phylogenetic analysis of green plant SGRs and bacterial SGR homologs

Protein sequences of SGRs and homologs were downloaded from the Phytozome v12.1 [24] (https://phytozome.jgi.doe.gov/pz/portal.html) and NCBI databases (https://www.ncbi.nlm.nih.gov/protein/). A total of 40 sequences were retrieved including 14 green plant SGRs, 3 archaeal and 23 bacterial SGR homologs representing a wide array of species with diverse homology [Supplementary Table 1]. The protein sequences of the green plant SGRs and their homologs were aligned using Clustal Omega with the default settings for multiple sequence alignment [25]. Identification and marking of the conserved residues in the MSA were performed in Jalview v2.11.1.4 [26]. The maximum likelihood phylogenetic tree was inferred using IQ-TREE v1.6.12 with 1000 bootstrap replicates in the ultrafast mode [27], [28]. The best-fitting amino acid substitution model for the dataset – WAG + I + G4 was applied automatically in the IQ-TREE server for phylogeny construction [29]. iTOL v6 was used for both visualization as well as generation of the figure [30].

### 2.2 Cloning of bacterial SGR homolog

*Anaerolineae* bacterium SM23_63 SGR homolog (AbSGR-h) encoded by KPK94580 with optimized codon usage for *E. coli* was artificially synthesized as previously reported [19]. AbSGR-h was amplified from the artificially synthesized DNA using the primer sets shown in Supplementary Table 2. Amplified DNA fragments are cloned into pET 30a (+) vectors (Novagen) containing a histidine-tag at the C terminus using the *Nde*I and *Xho*I sites through an in-fusion cloning system (Clontech). Several point mutations were introduced by PCR using primers as shown in Supplementary Table 2.

### 2.3 Expression and detection of recombinant proteins

The constructed plasmids for protein expression were introduced into *E. coli* BL21 (DE3). *E. coli* was grown and recombinant protein was expressed in an auto-induction medium (6 g Na_2_HPO_4_, 3 g KH_2_PO_4_, 20 g tryptone, 5 g yeast extract, 5 g NaCl, 6 mL glycerol, 5 g glucose, 2 g lactose, 100 mg kanamycin in 1 L) at 37 °C for 16 h with 120 rpm shaking [31]. After incubation, 1 mL of the cell was harvested by centrifuge at 20,000 g for 1 min. The pellet was suspended with 500 µL of BugBuster Protein Extraction Reagent (Millipore) with 0.1% Benzonase nuclease (Millipore). Crude supernatant was obtained by centrifugation at 20,000 g for 1 min. The whole cell lysate and crude supernatant were mixed with the same volume of sample buffer containing 125 mM Tris-HCl, pH 6.8, 4% (w/v) SDS, 10% (w/v) sucrose, and 5% (v/v) 2-mercaptoethanol. Mixtures were incubated at 95 °C for 1 min and 10 µL of the mixture was subjected to SDS-PAGE as previously reported [19]. Proteins were visualized by staining with Coomassie Brilliant Blue.

### 2.4 Recombinant protein purification

After induction of the recombinant protein, 100 mL of culture cells were harvested by centrifugation at 7,000 g for 5 min. The harvested cells were resuspended in buffer A (20 mM Na-phosphate pH 7.4, 100 mM NaCl, 20 mM imidazole) and disrupted by sonication (Branson Sonifier SFX250: output 8, duty cycle 20%) for 6 min in an ice bath. After sonication, dodecyl β-maltoside (βDM) was added to the final concentration of 0.05% (w/v) and incubated for 5 min at 25 °C. The cleared supernatant of cell lysate was obtained by centrifugation at 20,000 g for 10 min and then loaded onto a 5 mL HisTrap HP column (Cytiva) equilibrated with buffer A containing 0.05% βDM using an ÄKTAprime plus system (Cytiva). The recombinant proteins were eluted by buffer B (20 mM Na-phosphate pH 7.4, 100 mM NaCl, 500 mM imidazole, 0.05% βDM). To examine the purity of the protein, elution was mixed with the same volume of the sample buffer and 10 µL of the mixture was used for SDS-PAGE. Purified protein was analyzed by size exclusion chromatography using Sephacryl S-400R (Cytiva) equilibrated with buffer C (20 mM Na-phosphate pH 7.4, 100 mM NaCl, 0.05% βDM). The protein elution profile was monitored by absorbance at 280 nm. The molecular weight of AbSGR-h was evaluated by comparison to protein standards (Gel Filtration Calibration Kit LMW, Cytiva).

### 2.5 Enzymatic assay

The cell lysate prepared by suspending with BugBuster Protein Extraction Reagent as described above was used to perform enzymatic assay. One µL of chlorophyll *a* dissolved in DMSO (1 nmol µL^−1^) was mixed with 50 µL of cell lysate and incubated for 10 min at 25 °C. The reaction was stopped by adding 200 µL of acetone. After centrifuging at 20,000 g for 10 min, the supernatant was subjected to HPLC equipped with a fluorescence detector (RF- 20A, Shimadzu). The pigments were separated through a Symmetry C8 column (4.6 × 150 mm, Waters) with an eluent [methanol : acetonitrile : acetone (1:3:1 v:v:v)] at a flow rate of 1 mL min^−1^ at 40 °C. The elution profiles were monitored at fluorescence excitation/emission wavelengths of 410/680 nm.

### 2.6 Tertiary structure prediction and validation

*De novo* protein modelling of SGR protein from *Arabidopsis thaliana* (AtSGR1; Accession ID: AT4G22920.1) and an SGR homolog from *Anaerolineae* bacterium SM23_63 (AbSGR-h; Accession ID: KPK94580.1) were performed using trRosetta, which builds the tertiary structure based on direct energy minimizations with a restrained Rosetta [32]. The restraints include inter-residue distance and orientation distributions, predicted by a deep residual neural network. Out of the five models predicted for each protein, only the model with the best confidence as judged by the template modeling score (TM-score), developed by Xu and Zhang [33], was selected for further evaluation. TM-score can be used as an approximate but quantitative criterion for protein topology classification ([33]. The stereochemical quality of the predicted models after energy minimization in GROMACS 2018.1 [34] were assessed through Verify3D [35], PROCHECK [36] and ERRAT [37] in the Structural Analysis and Verification Server (SAVES) v. 5.0 server (https://servicesn.mbi.ucla.edu/SAVES/). The ProSA-web server (https://prosa.services.came.sbg.ac.at/prosa.php) was also used to validate the 3D models [38]. ProSA evaluates model quality in terms of a ‘Z’ score and provides a ‘Z’ score plot, where the predicted model is placed within experimental NMR and X-ray structure of equal residue length. PyMOL v. 2 was used for graphic modifications, visualization and preparing final illustrations [39]. Protein cavity detection was implemented in the CavityPlus web server (http://www.pkumdl.cn/cavityplus), which utilizes the 3D structural information as input to detect potential binding sites on the surface of a given protein structure [40]. Metal ion-binding site in the protein was predicted using the MIB server [41]. The ConSurf server (http://consurf.tau.ac.il/) was used to determine the functional regions in the modeled protein. It is a tool which analyzes the evolutionary dynamics of amino acid substitutions among homologous sequences and maps them onto the structure of the query protein [42]. Additionally, ConSeq v. 1.1, integrated in the ConSurf server, was used to identify the functionally and structurally important residues in the amino acid sequence of AbSGR-h [43]. Furthermore, five mutated monomeric structures of AbSGR-h (T29A, H32A, D34N, D62N and R26D + D114R, referred as the double mutant) were generated in PyMOL using the Mutagenesis wizard by selecting the most probable rotamers for each amino acid substitution.

### 2.7 Molecular dynamics simulation

Molecular dynamics simulation was performed on the predicted structure of the wild-type and five mutated monomers of the AbSGR-h protein using the GROMACS simulation package 2018.1. OPLS-AA/L all-atom force field was used to model the intramolecular protein interactions and the intermolecular interactions between the protein and solvent molecules [44]. Initially the energy of each system was minimized using 500 steps of the steepest descent algorithm followed by 20,000 steps of the Polak-Ribiere conjugate gradient method to remove the strain in the initial structure. The relaxed structure was immersed in a cubic box of extended simple point charge (SPC/E) water molecules with periodic boundary conditions in all directions. A minimum distance between the protein and the wall of the cell was set to 1 nm. Prior to energy minimization with periodic boundary conditions, each solvated system was neutralized by the addition of sodium and chloride ions.

MD simulation consists of equilibration and production phases. In the first stage of equilibration, the solutes (protein, counter ions) were fixed, and the solvent (water molecules) was equilibrated for 100 ps of MD at 200 K using an integral time step of 0.001 ps. During the equilibration phase, velocity was assigned to the atoms using Maxwell distribution. The system was coupled to the heat bath and heated to 300 K in a short run of 100 ps (0.001 ps time step) in which the system was allowed to relax in the new condition. This was followed by another short simulation of 100 ps with pressure coupling at 1 atm. Finally, the production phase of MD simulation was run keeping the temperature, pressure and number of molecules of the ensemble invariant. Production phase was continued up to 200 ns using 0.002 ps time step for each of the wild-type and mutated AbSGR-h proteins. The average structures for each monomer were obtained using the 200 ns trajectory of the MD production run. Subsequent analyses that include RMSD (Root Mean Square Deviation), RMSF (Root Mean Square Fluctuation) and radius of gyration (*Rg*) were performed using different programs of the GROMACS package on the 200 ns trajectory of the production run. The secondary structure content of the wild-type and mutant proteins along the production phase trajectory was computed using DSSP [45]. The webPSN v. 2 server [46] was utilized for the analysis of network of interacting amino acids wherein the average structure of the wild-type and mutant proteins were considered.

### 2.8 Protein-ligand docking study

The structure of chlorophyll *a* was retrieved from the KEGG LIGAND database (https://www.genome.jp/kegg/ligand.html) and was subjected to geometry optimization under the semi-empirical method in HyperChemTM 8.0.8 molecular modeling software (Hypercube Inc., Gainesville, FL, USA). Steepest descent followed by the Polak-Ribiere conjugate gradient algorithm was performed for energy optimization of chlorophyll *a* until convergence was reached. Open Babel was used for the interconversion of structures with different file formats [47]. Protein-ligand docking studies were carried out using AutoDock Vina v1.1.2 considering the average structures of wild-type and mutant AbSGR-h proteins obtained from respective MD simulations of 200 ns [48]. The pre-docking parameters were set using AutoDock Tools v4 with the addition of polar hydrogen atoms and Gasteiger charges to the protein molecule [49]. A grid box of 30 Å × 30 Å × 30 Å with grid spacing of 1 Å was set for docking. Hydrophilic and hydrophobic interactions in the docked conformations were visualized using PyMOL.

## 3 Results

### 3.1 Sequence comparison and phylogenetic analysis

**Fig. 2:**
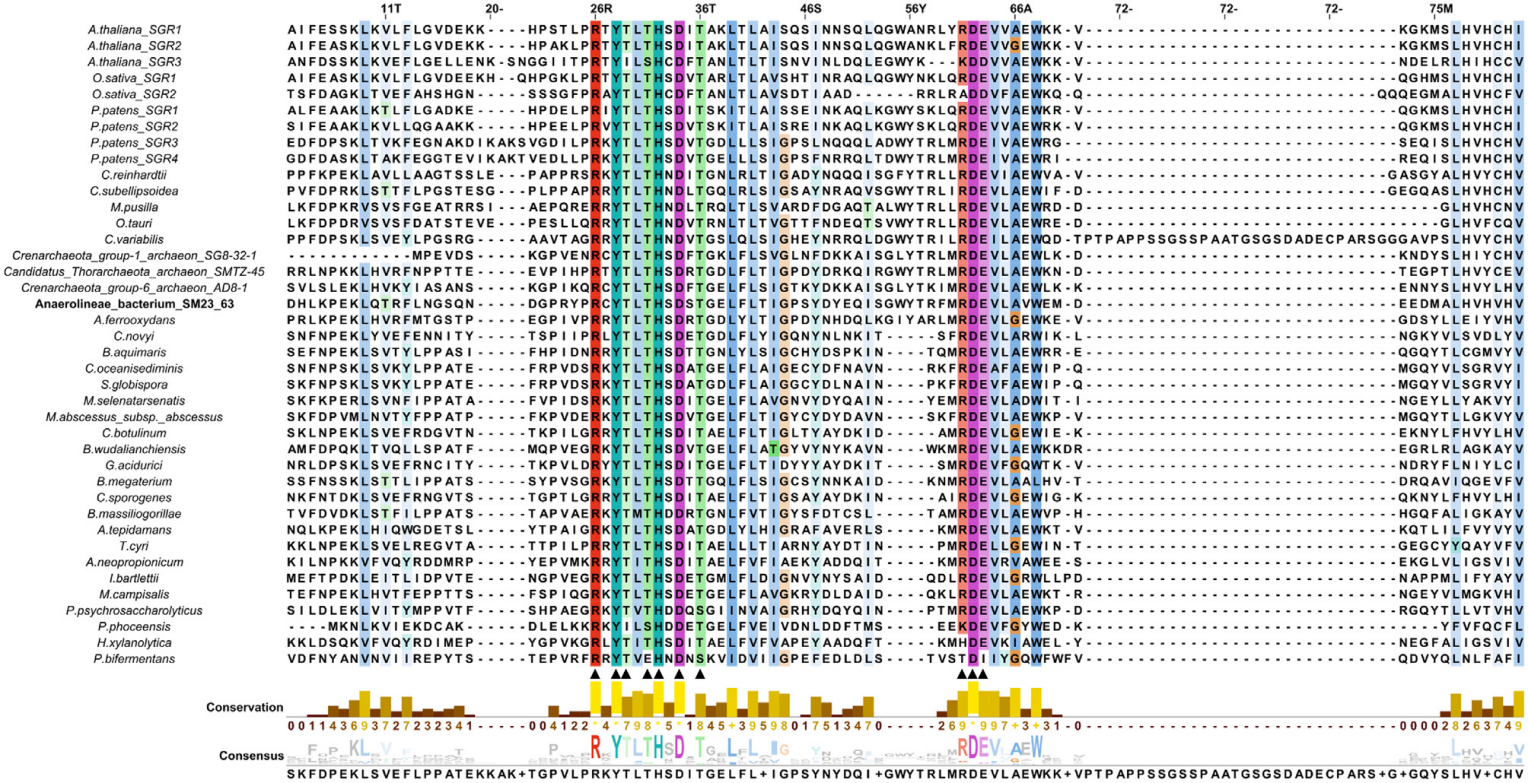

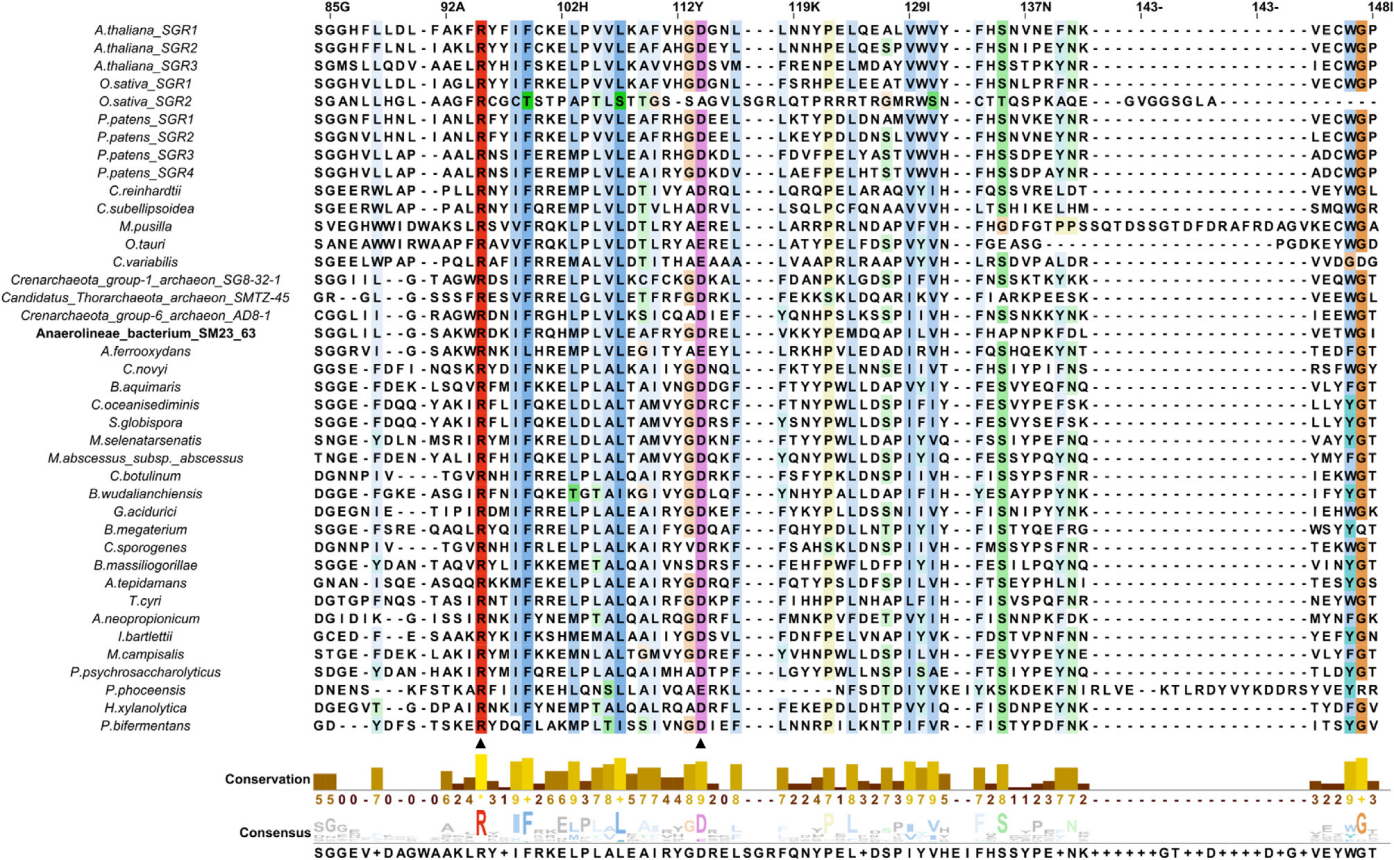
Multiple sequence alignment of green plant SGRs, archaeal and bacterial SGR homologs performed using Clustal omega. The alignment is coloured in the Clustalx format. Solid triangles denote residues that have been mutated in the subsequent biochemical experiments. Portion of the alignment displaying only conserved stretch of residues have been shown. (For interpretation of the references to color in this figure legend, the reader is referred to the web version of this article.)

Amino acid variability among 40 SGR and its homologs was observed from the analysis of the multiple sequence alignment that included 14 SGR sequences from green plants, 3 archaeal and 23 bacterial sequences as SGR homologs. Fig. 2 shows the alignment of the conserved region which depicts high sequence similarity between SGRs of photosynthetic eukaryotes and SGR homologs of archaea and bacteria. A total of 26 amino acid residues with conservation score above 90% were identified from the multiple sequence alignment. We also observed the presence of a motif similar to an incomplete metal-ion-dependent adhesion site (MIDAS) motif at 31–36 (T-H-S-D-S-T) for the SGR homolog from *Anaerolineae* bacterium SM23_63 shown in boldface in the multiple sequence alignment. A complete MIDAS motif usually consists of a consensus sequence (D-x-S-x-S… T… D) while an imperfect one is characterized either by the presence of conserved region 1 (D-x-S-x-S), without one or both of T and D, or those with conservative changes in region 1 with and without conservation of T and D [50]. Although the last T remains conserved in SGRs considered here, but for typical MIDAS motifs the T comes many amino acids after the conserved region 1. Structural studies of proteins containing this motif indicate that an imperfect MIDAS motif is also capable of binding metal ions. Although the presence of this motif had been reported in prokaryotic and plant chelatases [50], [51], the existence and role of the same in metal dechelatase are yet to be determined.

A Maximum-likelihood phylogenetic tree revealed a distinct clading pattern of SGR proteins across different life forms – ranging from archaea to green plants (Supplementary Figure 1). Though SGR proteins possess significant sequence similarity in their respective domains, SGRs did not intermix with its homologs in the phylogeny.

### 3.2 Protein expression and purification

Among all other bacterial SGR homologs studied here, SGR of *Anaerolineae* bacterium SM23_63 (AbSGR-h) is phylogenetically closer to that of the green plants. High expression level of the gene can be observed in *E. coli*. Interestingly, AbSGR-h shows much higher Mg-dechelating activity than *Arabidopsis* SGRs [19]. Furthermore, the genome of *Anaerolineae* hosts a single gene of SGR unlike genomes of other species that accommodate several homologous SGR genes.

**Fig. 3:**
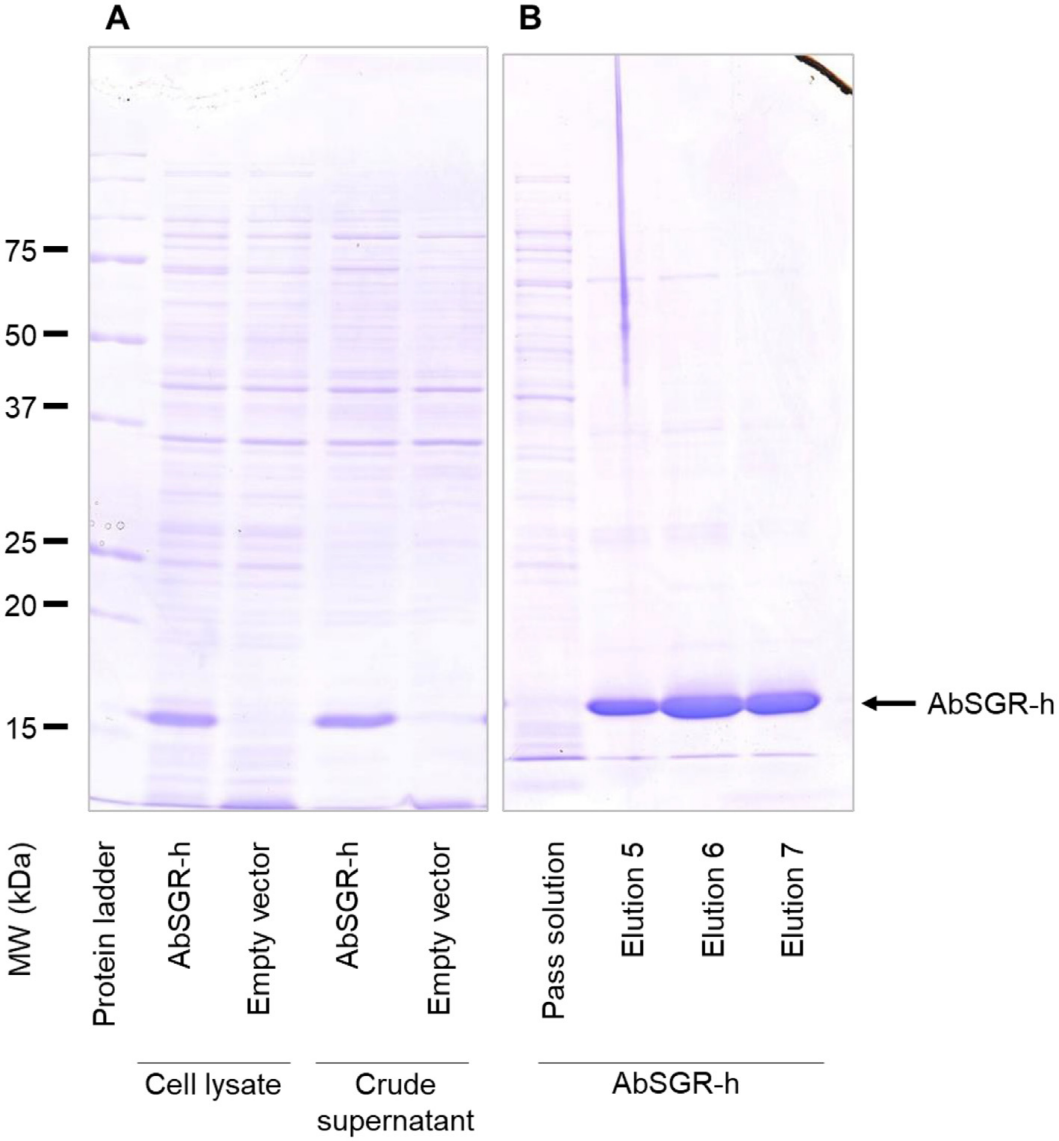
(A) Expression and (B) purification of the recombinant AbSGR-h. Histidine-tagged AbSGR-h was expressed in *E. coli* and purified by the nickel column.

The molecular weight and solubility of the expressed AbSGR-h protein was checked using SDS-PAGE (Fig. 3A). In the lysate of the AbSGR-h-expressing cells, the protein appeared as a single and prominent band corresponding to a molecular size of approximately 18 kDa. The band is absent in the cell lysate prepared from *E. coli* having empty vector indicating recombinant AbSGR-h to have been successfully expressed in *E. coli*. The soluble nature of AbSGR-h was confirmed from the presence of a clear band in the crude supernatant fraction. The protein was purified using a nickel column where the eluate in fractions 5–7 showed the maximum concentrations of the purified protein (Fig. 3B). The CBB-stained gel showed that the purity of AbSGR-h in elution is high.

### 3.3 Analysis of SGR mutants

Several mutants of AbSGR-h were constructed in this study to understand the effect of mutations on the structure and function of the protein. The mutations were carried out at conserved amino acid positions as found out from the multiple sequence alignment in Fig. 2. Altogether, eight charged amino acid residues (Arg26, His32, Asp34, Arg61, Asp62, Glu63, Arg95 and Asp114) and three threonine residues (Thr29, Thr31 and Thr36) were mutated. One conserved aromatic amino acid (Tyr28) was also changed. According to the modeled AbSGR-h structure (discussed later), an intramolecular electrostatic interaction between Arg26 and Asp114 was predicted, so we included an additional double mutation (R26D + D114R) by swapping their positions. Furthermore, to ensure that no steric repulsion occurs after the swapping, we substituted a non-conserved arginine at the 115th position with alanine, thereby, creating a triple mutant (R26D + D114R + R115A).

**Fig. 4:**
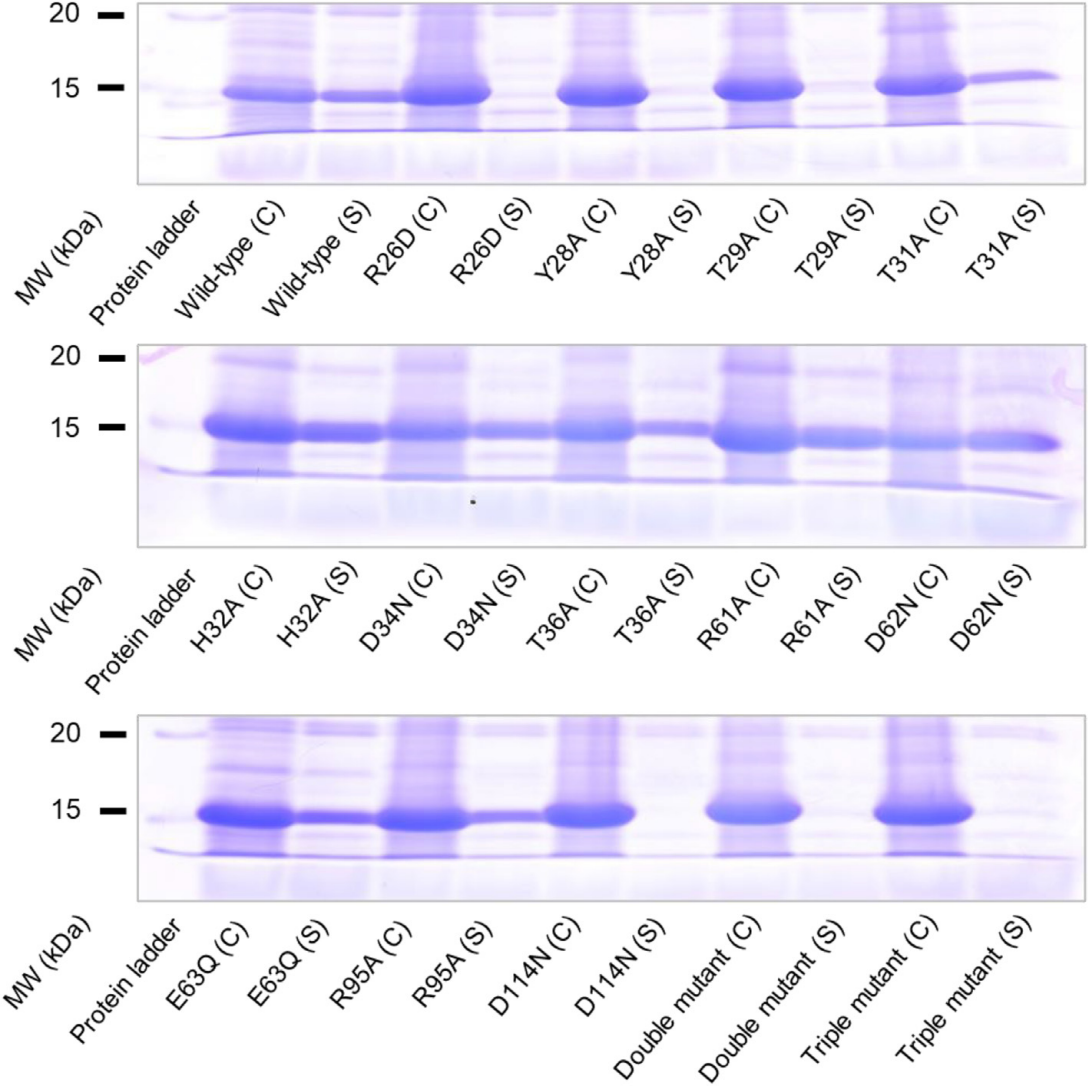
Examination of solubility of the expressed proteins. Wild-type and mutant AbSGR-h were expressed in *E. coli*. After lysis of *E. coli*, crude cell lysate (C) and the soluble fraction of cell lysate (S) were applied on SDS-PAGE.

After expression of SGR mutants in *E. coli*, solubility of the wild-type and mutant proteins was analyzed by running the soluble fraction in SDS-PAGE (Fig. 4). Expressed protein bands were observed in the crude cell lysate derived from *E. coli* having mutant constructs. Since solubility is intrinsically linked to the structural integrity of a protein, mutation in the sequence might disrupt the interaction network between the amino acids resulting in strong destabilization of the structure ultimately leading to the loss of solubility. The solubility of the SGR mutants – R26D, Y28A, T29A and D114N was significantly decreased when compared with the wild-type. The same was observed with the double mutant (R26D + D114R) with exchanged residue position and the triple mutant (R26D + D114R + R115A) with an additional mutation to avoid positively charged arginine in consecutive positions. Thus, we suggest that these residues play a key role in maintaining the conformation of the protein.

### 3.4 Enzymatic activity

**Fig. 5:**
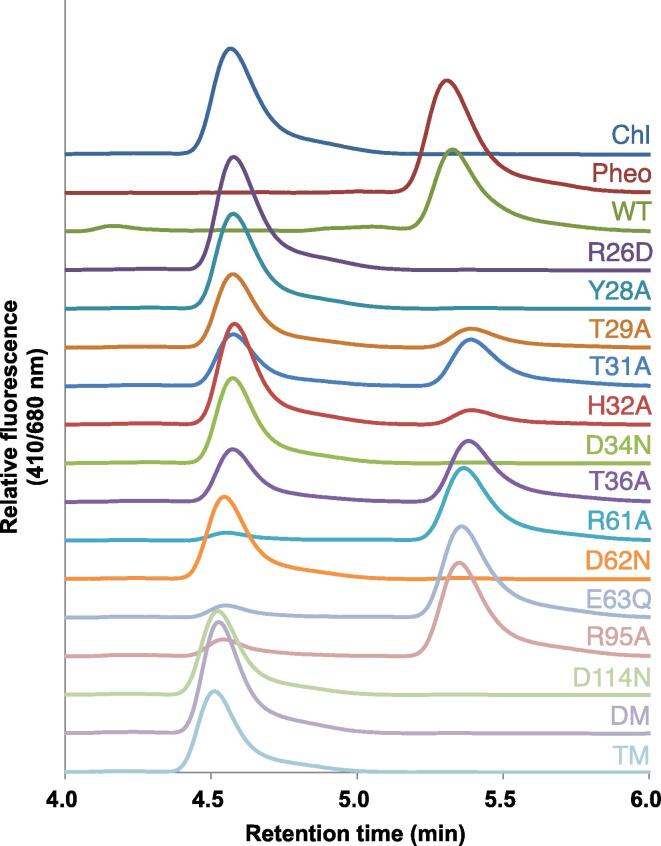
Determination of AbSGR-h activity. Chlorophyll *a* was incubated with crude cell lysate of *E. coli* expressing wild-type or mutant AbSGR-h. After incubation, pigments were extracted and analyzed by HPLC. Chlorophyll *a* and pheophytin *a* peaks have also been shown.

Enzymatic activity of AbSGR-h results in the removal of Mg^2+^ from chlorophyll *a* and produces pheophytin *a*. To assess the effect of the mutations on enzymatic activity of the wild-type and mutant proteins, cell lysates were incubated with chlorophyll *a*. Activity levels were evaluated based on the amounts of the product, pheophytin *a*, on HPLC profiles (Fig. 5). It may be emphasized that appearance of the pheophytin *a* peak is associated with the concomitant disappearance of the substrate chlorophyll *a*. Mutants R61A, E63Q and R95A along with the wild-type AbSGR-h showed high activity (major peak corresponding to pheophytin *a*), suggesting that these mutations does not affect the Mg-dechelating activity of the protein at all. AbSGR-h mutants T31A and T36A exhibited moderate activity whereas T29A and H32A mutation made the protein weakly active. Rest of the mutations *i.e.*, R26D, Y28A, D34N, D62N, D114N as well as double and triple mutations rendered the protein inactive. Both D34N and D62N showed no activity despite being soluble, implying their potential role in catalyzing the Mg-dechelatase reaction. The inactive nature of the remaining mutations can be attributed to their insolubility due to disruption of the protein structure.

### 3.5 Complex formation of wild-type and mutant AbSGR-h

**Fig. 6:**
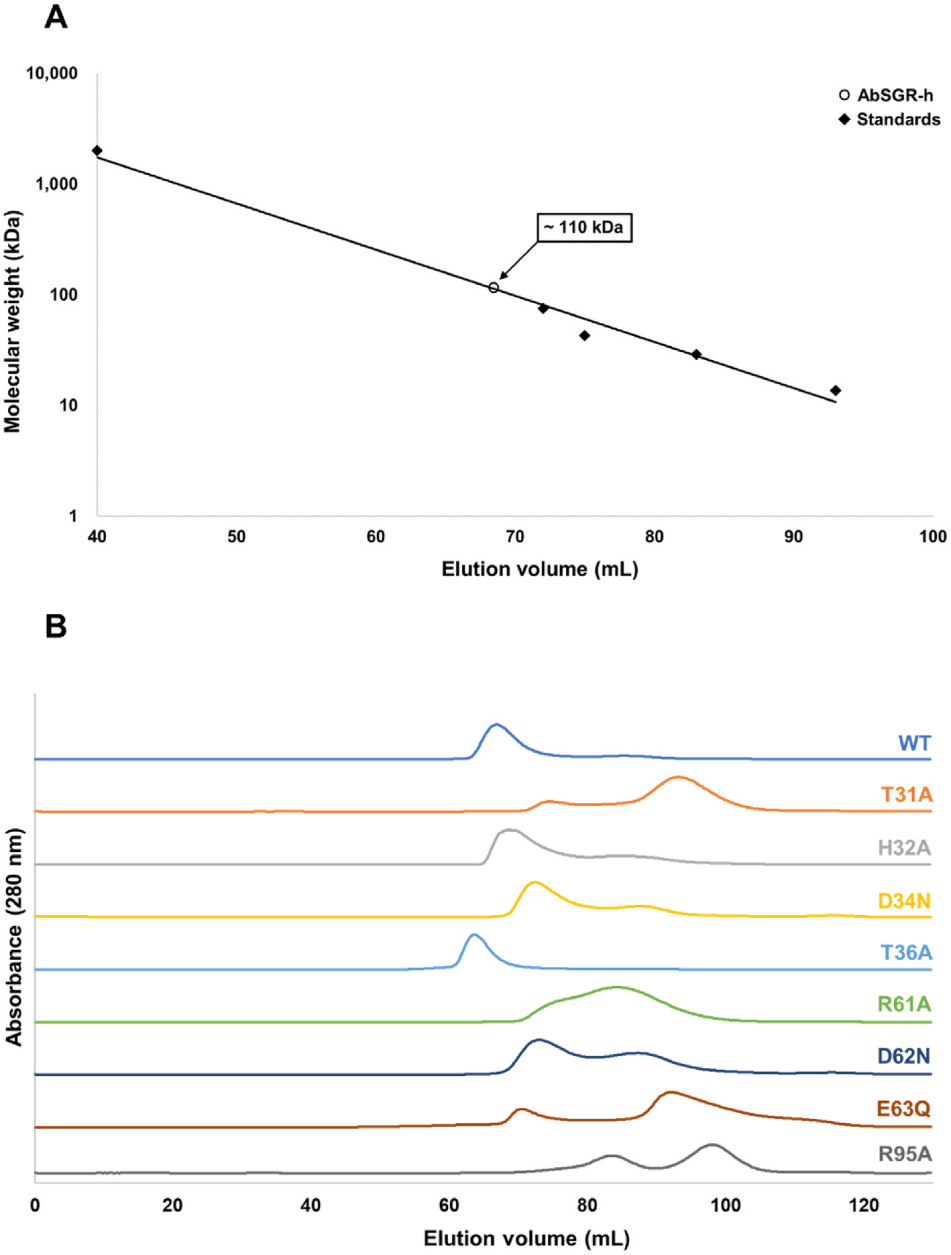
Determination of the molecular size of AbSGR-h complex. (A) Calibration curve of logarithm of molecular mass as a function elution volume. (B) Size exclusion chromatography profiles of AbSGR-h. Protein was monitored by the absorbance at 280 nm.

Some of the chlorophyll metabolizing enzymes form oligomeric complexes [52], [16]. Under the assumption that SGR may also form an oligomeric complex, size exclusion chromatography was performed. The apparent molecular weight of wild-type AbSGR-h was evaluated by comparison to globular protein standards with known molecular weights. Comparison of the single, major AbSGR-h peak with the calibration curve yielded a molecular weight of approximately 110 kDa (Fig. 6A). The apparent molecular weight of AbSGR-h monomer obtained from SDS-PAGE analysis revealed the apparent molecular weight of AbSGR-h monomer to be 18 kDa, implying the eluted wild-type protein to possibly exist as a hexameric complex.

Further, in order to determine the effect of amino acid substitutions on complex forming ability, size exclusion chromatography was carried out for individual soluble AbSGR-h mutants (Fig. 6B). Along with the wild-type protein, similar major peaks were observed for H32A, D34N, T36A and D62N mutants, suggesting presence of hexameric complexes in these mutants. Absence of a major peak at that position in case of the other mutants like T31A, R61A, E63Q and R95A indicates loss of the hexameric form upon mutation. However, the major peak positions differed slightly among proteins, probably because the system used for the analysis was not very stable and the flow rate of the solution could not be completely regulated. Interestingly, though R61A, E63Q and R95A mutants lost their hexameric form, they remain highly active suggesting that the formation of multimeric complex is not indispensable for the catalytic activity of SGR. However, the reason behind the multimeric conformation and its implication on the function of SGR remains elusive.

### 3.6 Predicted 3D structure of SGR and its homolog

**Fig. 7:**
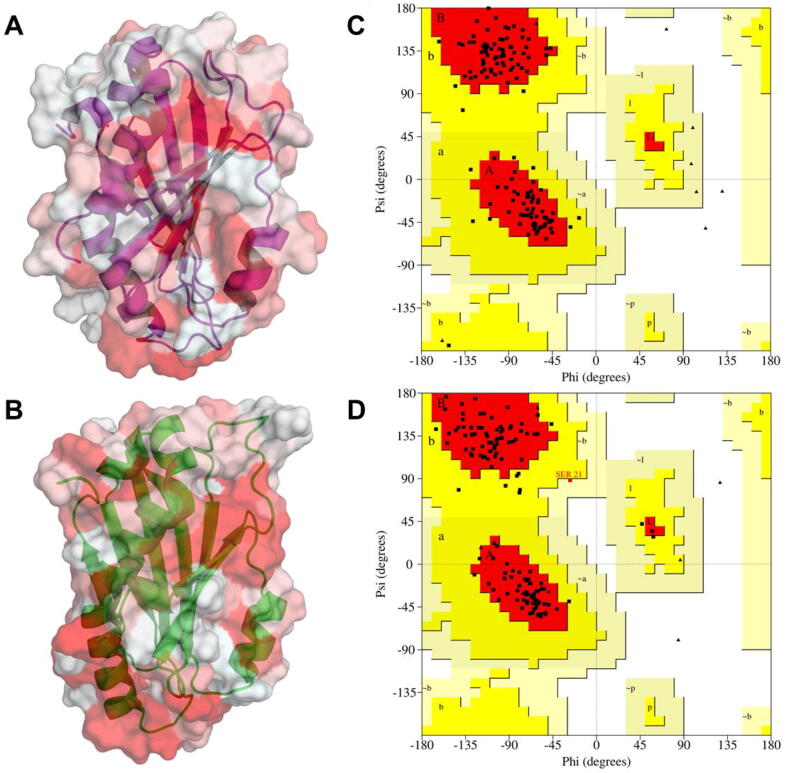
Hydrophobic surface and cartoon representation of the predicted three-dimensional structure of (A) AbSGR-h and (B) AtSGR1 where white and red color indicate hydrophilic and hydrophobic regions, respectively. Ramachandran plot showing the dihedral angle values for (C) AbSGR-h and (D) AtSGR1 is also given. (For interpretation of the references to color in this figure legend, the reader is referred to the web version of this article.)

Spatial location of the amino acid residues in the predicted protein tertiary structure might be insightful for a better understanding of the functioning of the dechelatase. A *de novo* approach for protein modelling was adopted due to lack of any experimentally determined structure for SGR or its homologs. We therefore modeled the structure of *Anaerolineae* SGR homolog (AbSGR-h) using trRosetta (Fig. 7A). The method is based on a deep residual-convolution network that is trained on native proteins to predict inter-residue distance and orientation. Among the five predicted models of AbSGR-h, the model with best confidence (estimated TM-score = 0.870) was selected for further analysis. Additionally, we also derived the tertiary structure of *Arabidopsis thaliana* SGR (AtSGR1) (Fig. 7B) using the same algorithm. The structure of AtSGR1 was modeled considering the amino acid sequence of its SGR domain only, as the entire sequence appears be too long to obtain a high confidence score. The TM-score for the modeled AtSGR1 domain was 0.840 whereas the same for the whole protein was 0.476.

Different quality evaluation programs such as PROCHECK, ERRAT and Verify 3D available online on the SAVES server, were used to assess the quality of the energy minimized modeled structures of AbSGR-h and AtSGR1. Ramachandran plots for both the protein structures demonstrated that the predicted models follow all the stereochemical properties with favourable phi (ϕ) and psi (ψ) values (Fig. 7C and 7D). Furthermore, protein quality assessment by ERRAT and Verify3D confirmed that the structures are highly accurate (Table 1). The ProSA analysis of AbSGR-h and AtSGR1 revealed a Z-score of −5.48 and −4.3, respectively, accommodating the modeled structures in the NMR zone and thus confirming their reliability (Supplementary Fig. 2).

**Table 1:** Model evaluation analyses for AbSGR-h and AtSGR1, obtained from the SAVES server.

	*Anaerolineae* SGR homolog (AbSGR-h)	*Arabidopsis* SGR(AtSGR)
	Ramachandran Plot Analysis	
Residues in most favoured regions	90.9%	91.2%
Residues in additional allowed regions	9.1%	8.0%
Residues in generously allowed regions	0.0%	0.8%
Residues in disallowed regions	0.0%	0.0%
	ERRAT	
Overall quality factor	81.618%	86.923%
	Verify3D	
Residues withaveraged 3D-1D score ≥ 0.2	94.77%	86.18%

The potential binding site on the surface of AbSGR-h protein was determined by CavityPlus. The residues constituting the predicted cavity are: T31, H32, S33, D34, S35, T36, E38, L39, F40, W55, R58, F59, M60, R61, D62 and R95 (Supplementary Fig. 3). Majority of the residues comprising the binding cavity were found to be conserved among SGRs. Additionally, we used ConSurf to find the evolutionary conservation score of each amino acid residue in AbSGR-h, where a score of less than 3 and more than 7 indicate variable and conserved residues, respectively (Supplementary Fig. 4A). The result of ConSeq analysis, which shows the degree of conservation as well as the structurally and functionally important residues along the sequence of AbSGR-h, is also depicted in Supplementary Fig. 4B.

### 3.7 Molecular dynamics simulation analysis

**Fig. 8:**
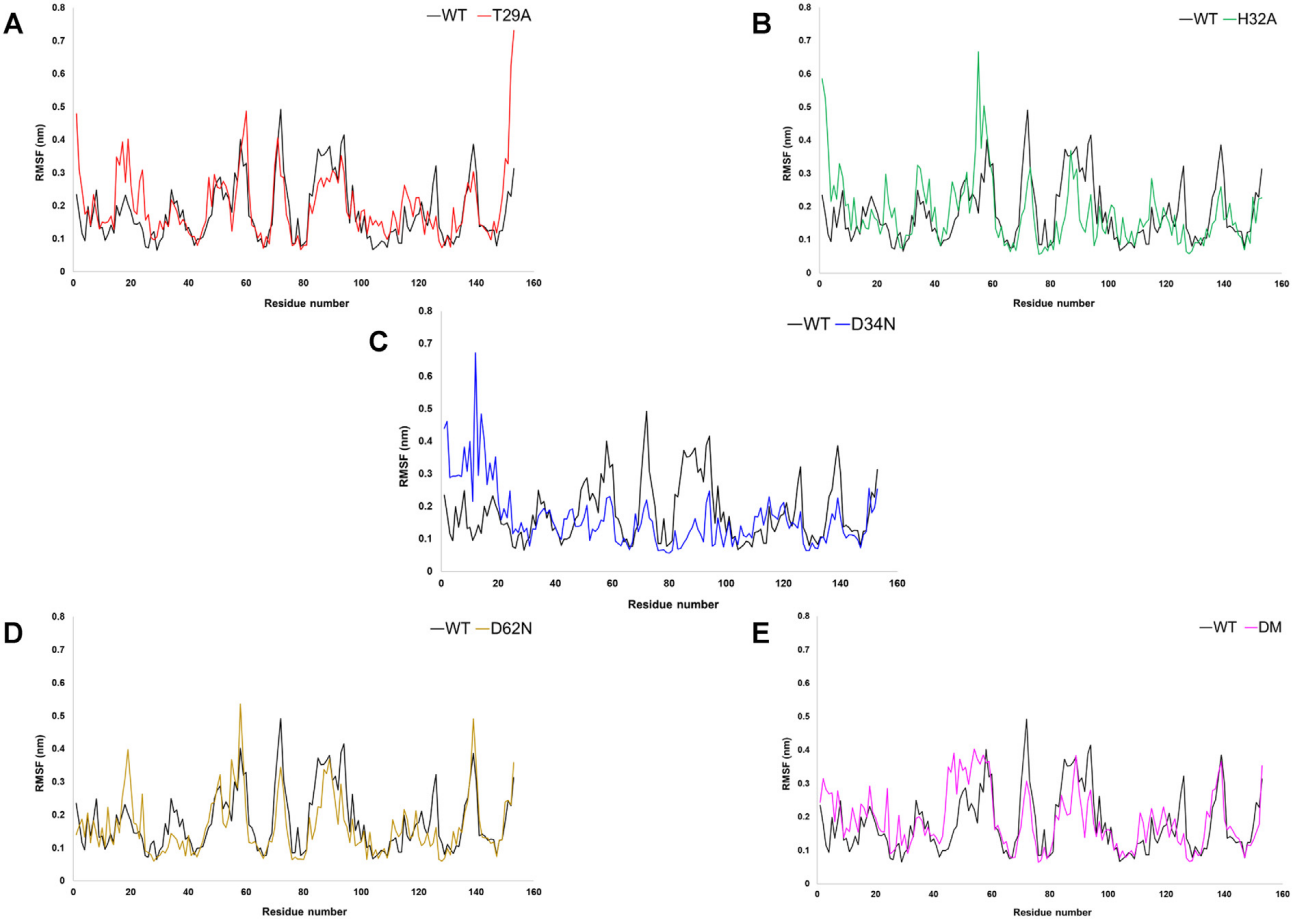
RMSF plots of each residue of wild-type and mutant AbSGR-h proteins over the 200 ns trajectory of the MD production run.

**Fig. 9:**
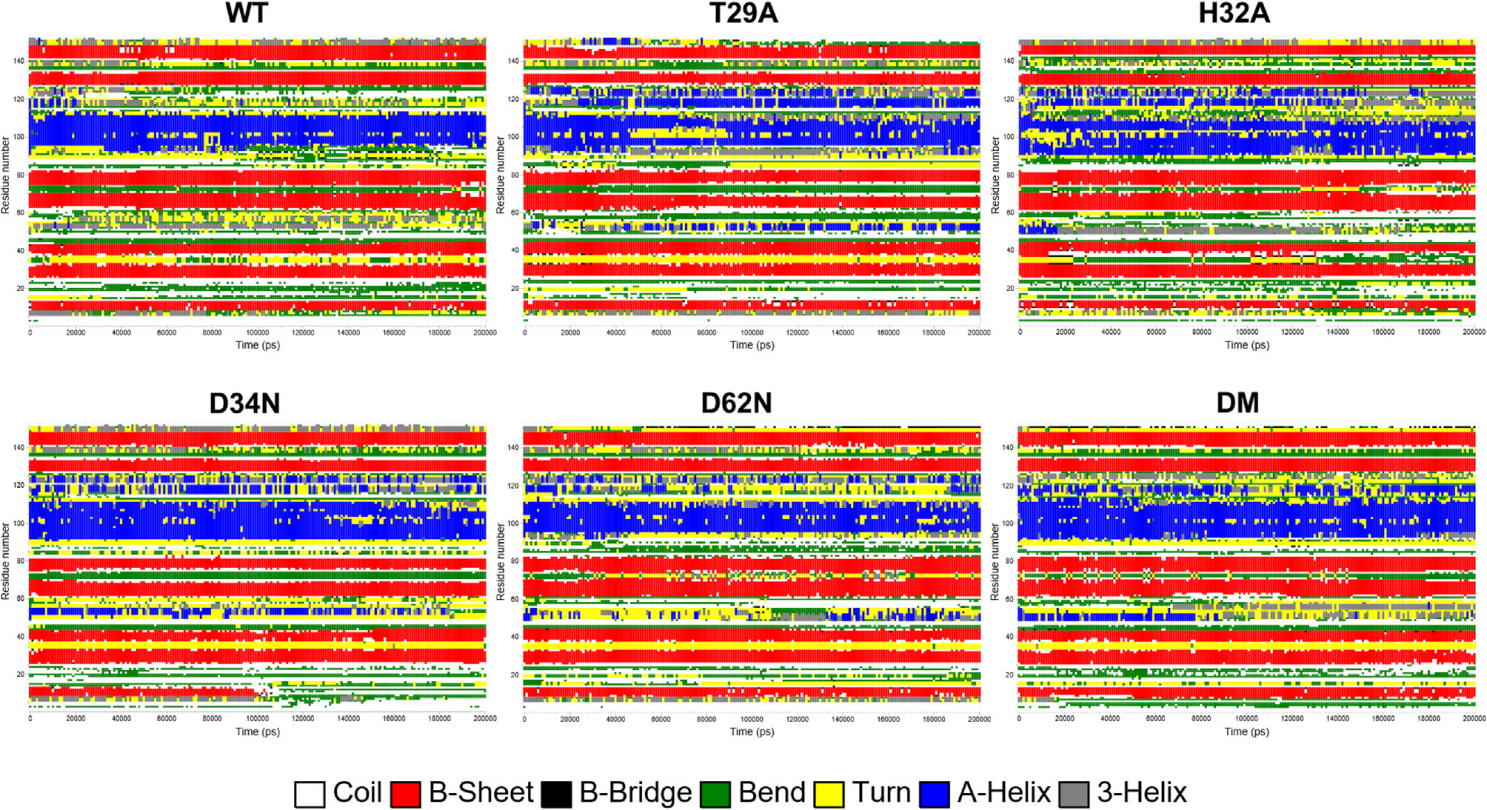
Time evolution of the secondary structures of wild-type and mutant AbSGR-h proteins over the 200 ns trajectory of the MD production run, determined using the DSSP method.

Molecular dynamics (MD) simulations were performed on wild-type AbSGR-h and five mutants (T29A, H32A, D34N, D62N and the R26D + D114R double mutant) for 200 ns to assess the structural stability and conformational dynamics of the predicted protein structure in its wild-type and mutated form. The time-dependent changes of RMSD of the backbone atoms for each protein was estimated considering the respective input structure for MD production run as reference. The RMSD plot of AbSGR-h and its mutants reflected convergence of the simulation, indicating the overall structural stability of all the monomers (Supplementary Fig. 5). The RMSF for individual residues for wild-type AbSGR-h and mutated monomers were computed to infer the residue specific flexibility, taking into account the respective input structure for MD production run as reference (Fig. 8). It is evident from the RMSF plot that the D34N mutant appear to be more rigid when compared to the wild-type and other mutated monomers. However, few residues pertaining to the N-terminus of the D34N and H32A mutant exhibited higher flexibility than the native protein. On the other hand, though the N-terminal and C-terminal ends of the T29A mutant showed more flexibility than the wild-type, both the wild-type and T29A monomers displayed similar fluctuation patterns from the 29th residue onwards. We also calculated the radius of gyration (*Rg*) which is a measure of the compactness of a protein. It is evident from the invariant *Rg* values (Supplementary Fig. 6) that none of the mutations grossly disrupt the monomeric structure of the protein. Further information on the structural flexibility for both the wild-type and mutant protein models are offered by the analysis of time-dependent secondary structure fluctuations (Fig. 9), calculated using the DSSP algorithm in GROMACS. It is interesting to note that once more the mutant D34N shows variation in the time evolution of the secondary structural elements. Out of the seven β-strands, the one at the N-terminal end gets disrupted after 100 ns of simulation. Amino-acid network analysis revealed R26 to be a hub residue interacting with six other residues, a reason that can be attributed to why the R26D mutant became insoluble. It is to be noted that even if the ionic interaction remains undisturbed in the double mutant (where the residue pair has been swapped), the replacement of R26 with D (an amino acid with shorter side chain) renders it incapable of being a hub residue thus disrupting all interactions with other amino acids, leading to insolubility. Likewise, the network analysis also revealed Y28 and T29 to interact with two and three other residues of AbSGR-h respectively, implying their importance in imparting the native structure of the protein. Mutation (Y28A or T29A) disturbs these interactions that in turn disrupts the native structure which probably is the cause of the observed insolubility.

### 3.8 Molecular docking analysis

**Fig. 10:**
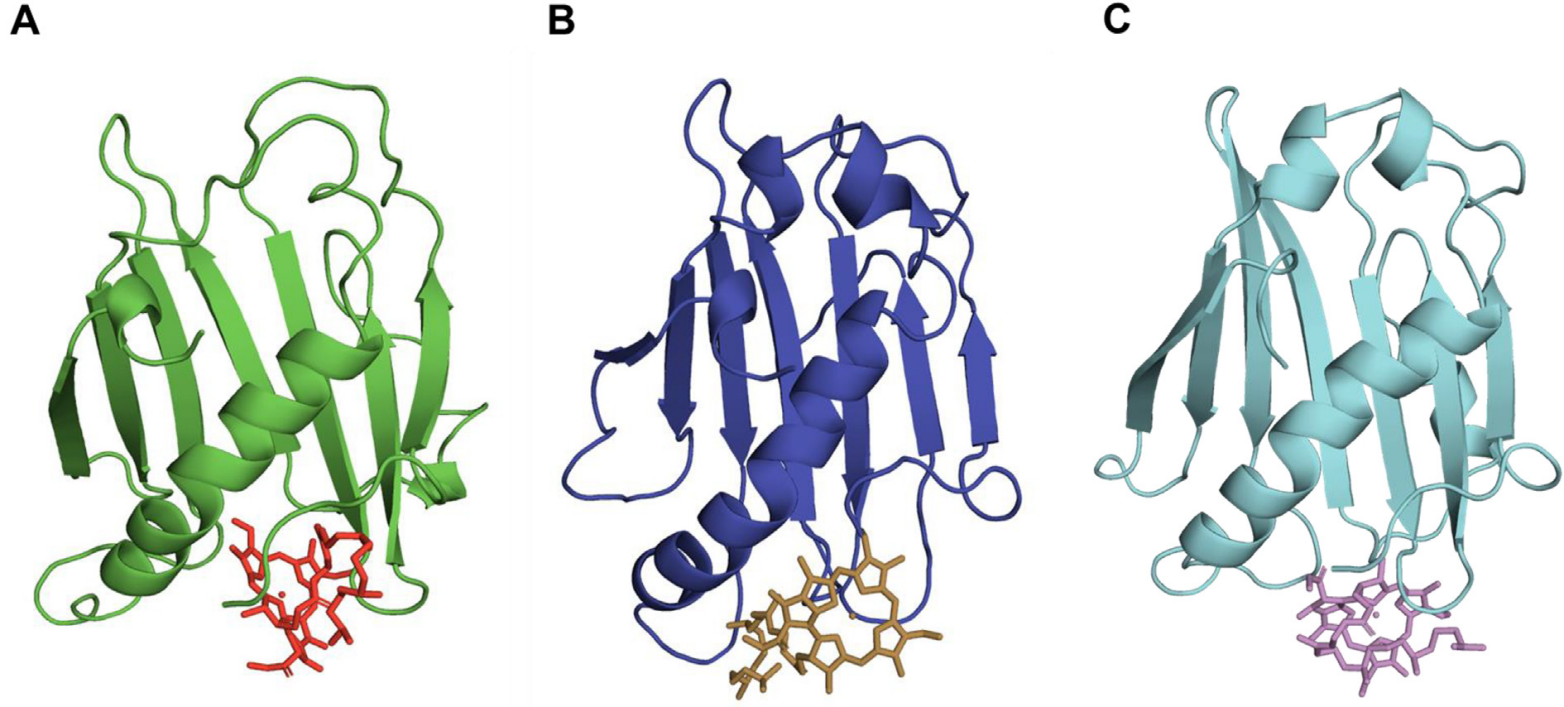
Docked structure of chlorophyll *a* with (A) wild-type, (B) H32A mutant and (C) D62N mutant AbSGR-h proteins. The average structure of wild-type and mutant monomers, obtained from the MD simulations of 200 ns, were docked with chlorophyll *a* using AutoDock Vina. The results of the docking study corroborates with those obtained from the enzyme activity analysis where the wild-type, H32A mutant and D62N mutant proteins showed high, negligible and no activity, respectively.

For docking analysis, energy-optimized free chlorophyll *a* was used as ligand of SGR. The actual substrate of SGR in the chloroplast is chlorophyll *a* bound to chlorophyll-protein complexes that are embedded in the thylakoid membrane [53]. Docking studies of chlorophyll *a* with the wild-type and mutated AbSGR-h proteins were carried out using two settings. In the first instance, the grid box was set large enough to cover the entire protein structure while in the second setting, we opted for a specific grid box with a size of 30 Å × 30 Å × 30 Å that was centered around the active site of the protein. We presumed D34 and D62 residues as the key components of the active site since biochemical experiments revealed that mutations of these two amino acids rendered the protein catalytically inactive despite being soluble. Furthermore, protein cavity prediction analysis by CavityPlus implicated the importance of these two aspartate residues. In addition, D34 was found to be a part of an incomplete MIDAS motif as observed from the multiple sequence alignment. The docked conformation of wild-type AbSGR-h revealed interaction of the ligand with H32 and D34 (Fig. 10A). However, despite being in the vicinity of the ligand, D62 did not show any interaction with the ligand. In both of the docking settings, chlorophyll *a* did not bind to the active site of the mutants T29A, D34N, D62N and double mutant. For the H32A mutant, although the ligand was found to interact with D34, the binding pose was different from that of the wild-type complex (Fig. 10B). Interestingly, enzymatic assay showed that H32A mutation made the protein weakly active, a fact that can be attributed to the altered binding state of the ligand with this mutated monomer. Furthermore, the result of docking analysis with the D62N mutant is consistent with the corresponding observation of the biochemical study (Fig. 5), which revealed that this residue is essential for the catalytic activity of the enzyme. The involvement of D62 in binding and/or activity is shown from the docked D62N-chlorophyll *a* complex (Fig. 10C), wherein the ligand orientation has changed from its wild-type counterpart (Fig. 10A).

## 4 Discussion

Despite technological advancement and sincere attempts, there remains a huge gap between the number of known sequences and experimentally derived structures available in the Protein Data Bank (PDB), highlighting the difficulties of structure elucidation by experimental methods like X-ray crystallography, nuclear magnetic resonance (NMR) spectroscopy and cryo-electron microscopy [54]. In recent times, protein tertiary structure prediction by deep learning-based methods made it possible to generate complete and accurate models of proteins that lack homologs in PDB. Our study provides the first three-dimensional structure of SGR, predicted by deep learning method using trRosetta. The quality and stability of the predicted AbSGR-h structure was probed by different protein quality evaluation programs and molecular dynamics simulation was also carried out with the model. We also compared the predicted structure with that obtained from the very recently developed RoseTTAFold tool [55] and found the RMSD (based on Cα-atoms) to be 1.2 Å.

The physiological relevance of bacterial SGR homologs remain unknown. Therefore, common and distinct characters between plant SGRs and bacterial SGR homologs have not been determined yet. Nevertheless, we considered working on a bacterial SGR homolog as a representative of the chlorophyll degrading enzyme of green plants for several reasons. The *Anaerolineae* SGR homolog (AbSGR-h) shares substantial functional similarity with the *Arabidopsis thaliana* SGR-Like (AtSGRL) protein, as evident from [19], where both of the proteins catalyze Mg dechelation with similar efficacy. The high sequence similarity among plant and bacterial SGR homologs, as seen in Fig. 2, indicate that the structurally and functionally important residues determined for a bacterial SGR are of equal importance to that of the green plant SGRs. Moreover, Asp107 and Asp132 of AtSGRL protein corresponds to Asp34 and Asp62 of AbSGR-h, respectively. Mutation of these aspartates in both the organisms exhibited loss of activity, indicating their catalytic role in the Mg dechelation reaction (data not shown for AtSGRL). Additionally, phylogenetic analysis revealed that AbSGR-h is close to plant SGRs (Supplementary Fig. 1), suggesting that they are functionally related. Furthermore, our study showed that the predicted structures of AbSGR-h and *Arabidopsis* SGR1 are highly similar (Fig. 7). Therefore, it can be stated that the reaction mechanism of plant SGRs and bacterial SGR homologs in terms of Mg extraction from chlorophyll may be similar, despite differences in their substrate specificity [19].

**Fig. 11:**
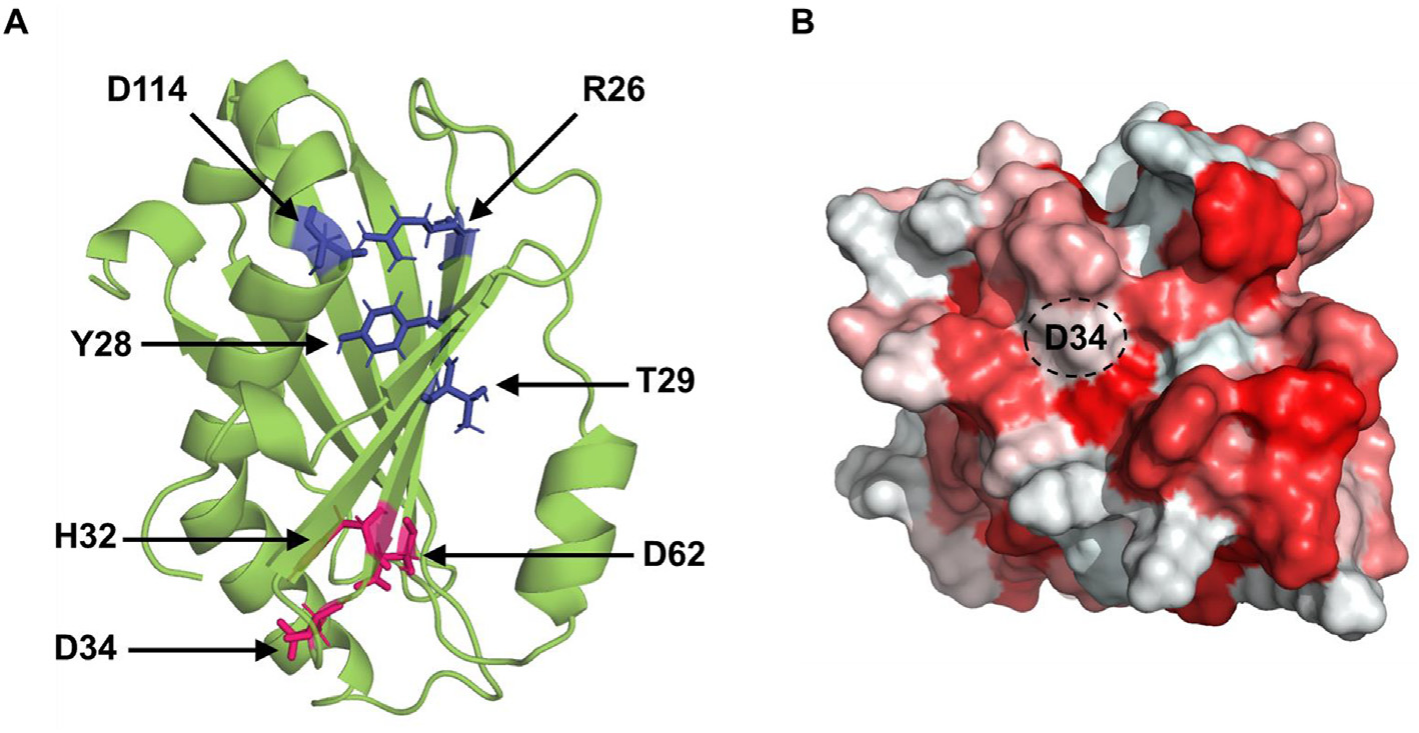
(A) Structure of AbSGR-h showing structurally and functionally important residues. Side chain of amino acids responsible for maintaining structure of the protein are marked in blue while those involved in catalysis are marked in red. (B) Hydrophobic patch surrounding D34 shown in the hydrophobicity surface representation of AbSGR-h. White and red color indicate hydrophilic and hydrophobic regions, respectively. (For interpretation of the references to color in this figure legend, the reader is referred to the web version of this article.)

SDS-PAGE analysis of the expressed AbSGR-h protein revealed the molecular weight of the monomer to be ∼ 18 kDa and size exclusion chromatography indicated that the recombinant protein may exist as a hexamer. Several AbSGR-h mutants were created by substituting conserved amino acid residues to determine their structural and functional significance (Table 2). Single mutations at R26, Y28, T29 and D114 made the protein insoluble, suggesting them to play an important role in the structural maintenance of the protein (Fig. 11A). These amino acids were found relatively close to each other spatially and remained buried in the predicted structure. In the model, R26 and D114 form an ionic bond that is lost when these amino acids were exchanged. Supporting data from amino acid network analysis of the protein structure revealed R26 to be a hub residue, that is engaged in different kinds of interaction with six other residues including D114. Exchange of R26 and D114 leads to the disruption of the other five interactions that appear to be essential for the ionic interaction to occur. Thus, for all the three mutations, R26D, D114N and R26D-D114R, disruption of the ionic bond in the native protein structure leaves the molecule insoluble. Similarly, network analysis showed that Y28 and T29 interact with two and three other residues respectively in the wild-type structure, all of which get disrupted upon mutation. This interruption probably leads to insolubility of the mutant forms, as evident from the biochemical analysis. Mutations of two specific aspartates – D34N and D62N made the protein inactive without affecting its solubility and ability to form multimeric complex (Fig. 11A). Incidentally, D34 is present within an incomplete MIDAS motif, the functional role of which is to dechelate ions. Docking of the Mg^2+^ to the predicted wild-type structure using the MIB server, displayed interaction of the ion with D62 suggesting potential catalytic role of D62 in the Mg-dechelatase enzyme. Mutation in T31, R61, E63, and R95 resulted in the destruction of the multimeric complex.

**Table 2:** Summary of the activity level and solubility of the wild-type and mutant AbSGR-h proteins.

AbSGR-h	Activity level	Solubility
Wild-type	Highly active	Soluble
R26D	Inactive	Insoluble
Y28A	Inactive	Insoluble
T29A	Inactive	Insoluble
T31A	Moderately active	Soluble
H32A	Weakly active	Soluble
D34N	Inactive	Soluble
T36A	Moderately active	Soluble
R61A	Highly active	Soluble
D62N	Inactive	Soluble
E63Q	Highly active	Soluble
R95A	Highly active	Soluble
D114N	Inactive	Insoluble
R26D + D114R (Double mutant)	Inactive	Insoluble
R26D + D114R + R115A (Triple mutant)	Inactive	Insoluble

Conformational dynamics at the monomer level was analyzed through molecular dynamics simulations of 200 ns carried out for the wild-type and five mutant proteins. Time-dependent changes of RMSD and invariant Rg values revealed the structural stability of the wild-type and mutant protein forms. Interestingly, the D34N mutant showed overall less flexibility than the wild-type and distortion of an N-terminal β-strand in the time evolution of secondary structural elements when analyzed by RMSF and DSSP, respectively. Molecular docking analysis displayed interaction of D34 with chlorophyll *a*, implying its importance once more in the catalytic activity of the enzyme. It is to be noted that the side chain of D34 is exposed to the surface of the predicted structure and residues surrounding D34 form a hydrophobic patch, an environment appropriate for interaction of chlorophyll *a* with the protein (Fig. 11B).

Central Mg^2+^ of chlorophyll *a* is held by two N atoms of the tetrapyrrole structure. SGR catalyzes Mg extraction from chlorophyll *a*, resulting in incorporation of two protons into the chlorin ring to produce pheophytin *a*. Since the catalytic mechanism of SGR remains unknown, two hypotheses can be proposed. The first one is similar to the reactions observed under acidic condition. The electrophilic attack of protons to the core N atoms of chlorophyll remove Mg^2+^, leading to the formation of pheophytin. As acidic amino acid residues can serve as proton donors, D34 can be considered as a potential residue involved in the dechelation reaction. On the other hand, formation of coordinate bond between an electronegative atom and central Mg^2+^ of chlorophyll destabilizes the Mg-N (pyrrole) interaction. Once this complex is formed, the Mg ion may be readily replaced with protons. Considering the optimum pH of SGR to be neutral [20] and the D34 residue to be present at the surface of the predicted structure, its de-protonated side chain can serve as a candidate to provide electronegative O for coordination with Mg^2+^.

In conclusion, by combining biochemical analysis and structural prediction of the SGR homolog from *Anaerolineae*, we provide the first structural insights into the SGR protein family. It will serve as a basis for further investigation of its reaction mechanism, functional analysis and other aspects such as inhibitor screening and/or evolutionary studies.

## CRediT authorship contribution statement

**Debayan Dey:** Conceptualization, Methodology, Investigation. **Dipanjana Dhar:** Formal analysis, Data curation. **Helena Fortunato:** Supervision. **Daichi Obata:** Investigation, Visualization. **Ayumi Tanaka:** Supervision. **Ryouichi Tanaka:** Supervision, Writing - review & editing. **Soumalee Basu:** Formal analysis, Supervision, Writing - review & editing. **Hisashi Ito:** Investigation, Supervision, Writing - review & editing.

## Declaration of Competing Interest

The authors declare that they have no known competing financial interests or personal relationships that could have appeared to influence the work reported in this paper.
