# Supplementary material for: Insights into the structure and function of the rate-limiting enzyme of chlorophyll degradation through analysis of a bacterial Mg-dechelatase homolog
Source: Comput Struct Biotechnol J. 2021 Sep 23;19:5333–47. doi: 10.1016/j.csbj.2021.09.023 (PMC8531759; doi:10.1016/j.csbj.2021.09.023)
Supplement: Supplementary data 1 [file mmc1.docx]

**Supplementary Material**

**Supplementary Table 1:** Details of SGRs and its homologs used for multiple sequence alignment and phylogenetic tree construction

| **Organism** | **Accession ID** | **Database** | **Taxon** |
| --- | --- | --- | --- |
| *Arabidopsis thaliana* | AT4G22920.1 | Phytozome | Green plant |
| *Arabidopsis thaliana* | AT4G11910.1 | Phytozome | Green plant |
| *Arabidopsis thaliana* | AT1G44000.1 | Phytozome | Green plant |
| *Oryza sativa* | LOC_Os09g36200.1 | Phytozome | Green plant |
| *Oryza sativa* | LOC_Os04g59610.1 | Phytozome | Green plant |
| *Physcomitrella patens* | Pp3c17_23030V3.1 | Phytozome | Green plant |
| *Physcomitrella patens* | Pp3c3_28140V3.1 | Phytozome | Green plant |
| *Physcomitrella patens* | Pp3c20_6290V3.1 | Phytozome | Green plant |
| *Physcomitrella patens* | Pp3c8_17510V3.1 | Phytozome | Green plant |
| *Chlamydomonas reinhardtii* | Cre12.g487500.t1.1 | Phytozome | Green plant |
| *Coccomyxa subellipsoidea* C-169 | XP_005650112.1 | NCBI | Green plant |
| *Micromonas pusilla* CCMP1545 | XP_003060977.1 | NCBI | Green plant |
| *Ostreococcus tauri* | XP_022840207.1 | NCBI | Green plant |
| *Chlorella variabilis* | XP_005850408.1 | NCBI | Green plant |
| miscellaneous Crenarchaeota group-1 archaeon SG8-32-1 | KON32503.1 | NCBI | Archaea |
| *Candidatus Thorarchaeota* archaeon SMTZ-45 | KXH70069.1 | NCBI | Archaea |
| miscellaneous Crenarchaeota group-6 archaeon AD8-1 | KON32872.1 | NCBI | Archaea |
| *Anaerolineae* bacterium SM23_63 | KPK94580.1 | NCBI | Bacteria |
| *Alicyclobacillus ferrooxydans* | WP_054971308.1 | NCBI | Bacteria |
| *Clostridium novyi* | WP_039245490.1 | NCBI | Bacteria |
| *Bacillus aquimaris* | WP_071617569.1 | NCBI | Bacteria |
| *Cytobacillus oceanisediminis* | WP_019379967.1 | NCBI | Bacteria |
| *Sporosarcina globispora* | WP_053435952.1 | NCBI | Bacteria |
| *Mesobacillus selenatarsenatis* | WP_041965262.1 | NCBI | Bacteria |
| *Mycobacteroides abscessus* subsp. *abscessus* | SHT39353.1 | NCBI | Bacteria |
| *Clostridium botulinum* sp. CDC54075 | WP_024931542.1 | NCBI | Bacteria |
| *Bacillus wudalianchiensis* | WP_065409936.1 | NCBI | Bacteria |
| *Gottschalkia acidurici* | WP_041701757.1 | NCBI | Bacteria |
| *Bacillus megaterium* | WP_013082933.1 | NCBI | Bacteria |
| *Clostridium sporogenes* | WP_058008982.1 | NCBI | Bacteria |
| *Bacillus massiliogorillae* | WP_042348482.1 | NCBI | Bacteria |
| *Anoxybacillus tepidamans* | WP_027408343.1 | NCBI | Bacteria |
| *Thalassobacillus cyri* | WP_093042251.1 | NCBI | Bacteria |
| *Anaerotignum neopropionicum* | WP_066089690.1 | NCBI | Bacteria |
| *Intestinibacter bartlettii* | WP_082421703.1 | NCBI | Bacteria |
| *Mesobacillus campisalis* | WP_046522531.1 | NCBI | Bacteria |
| *Peribacillus psychrosaccharolyticus* | WP_040373082.1 | NCBI | Bacteria |
| *Peptoniphilus phoceensis* | WP_062552496.1 | NCBI | Bacteria |
| *Hungatella xylanolytica* | WP_104438939.1 | NCBI | Bacteria |
| *Paraclostridium bifermentans* | WP_021432481.1 | NCBI | Bacteria |

**Supplementary Table 2: Primer sequences for cloning**

|  | **Forward (5' → 3')** | **Reverse (5' → 3')** |
| --- | --- | --- |
| **WT** | AAGGAGATATACATATGGATCATCTGAAACCGGAG | GGTGGTGGTGCTCGATTTTGTAATCGCGCAGAATG |
| **R26D** | TTATCCGGATTGCTATACCCTGACCCACAG | TATAGCAATCCGGATAACGCGGACCATCGT |
| **Y28A** | TCCGCGCTGCGCCACCCTGACCCACAGCGA | GGGTCAGGGTGGCGCAGCGCGGATAACGCG |
| **T29A** | GCGCTGCTATGCCCTGACCCACAGCGACAG | TGGGTCAGGCCATAGCAGCGCGGATAACGC |
| **T31A** | CTATACCCTGGCCCACAGCGACAGCACCGG | TGTCGCTGTGGCCCAGGGTATAGCAGCGCG |
| **H32A** | CCTGACCGCCAGCGACAGCACCGGTGAACT | TGTCGCTGCCGGTCAGGGTATAGCAGCGCG |
| **D34N** | CCACAGCAATAGCACCGGTGAACTGTTTCT | CGGTGCTATTGCTGTGGGTCAGGGTATAGC |
| **T36A** | CAGCGACAGCGCCGGTGAACTGTTTCTGAC | GTTCACCGCCGCTGTCGCTGTGGGTCAGGG |
| **R61A** | CTTTATGGCCGATGAAGTGCTGGCCGTGTG | CTTCATCGCCCATAAAGCGGGTGTACCAGC |
| **D62N** | TTTATGCGCAATGAAGTGCTGGCCGTGTGG | GCACTTCATTGCGCATAAAGCGGGTGTACC |
| **E63Q** | GCGCGATCAGGTGCTGGCCGTGTGGGAGAT | CCAGCACCTGATCGCGCATAAAGCGGGTGT |
| **R95A** | CAAATGGGCCGATAAAATCTTCCGCCAGCA | TTTTATCGCCCCATTTGGCGCTGCCCAGAA |
| **D114N** | TTATGGCAATCGCGAGCTGGTGAAGAAGTA | GCTCGCGATTGCCATAACGAAAGGCTTCCA |
| **D114R** | TTATGGCCGCCGCGAGCTGGTGAAGAAGTA | GCTCGCGGCGGCCATAACGAAAGGCTTCCA |
| **D114R-R115A** | TTATGGCCGCGCCGAGCTGGTGAAGAAGTATCC | CCAGCTCGGCGCGGCCATAACGAAAGGCTTCCA |


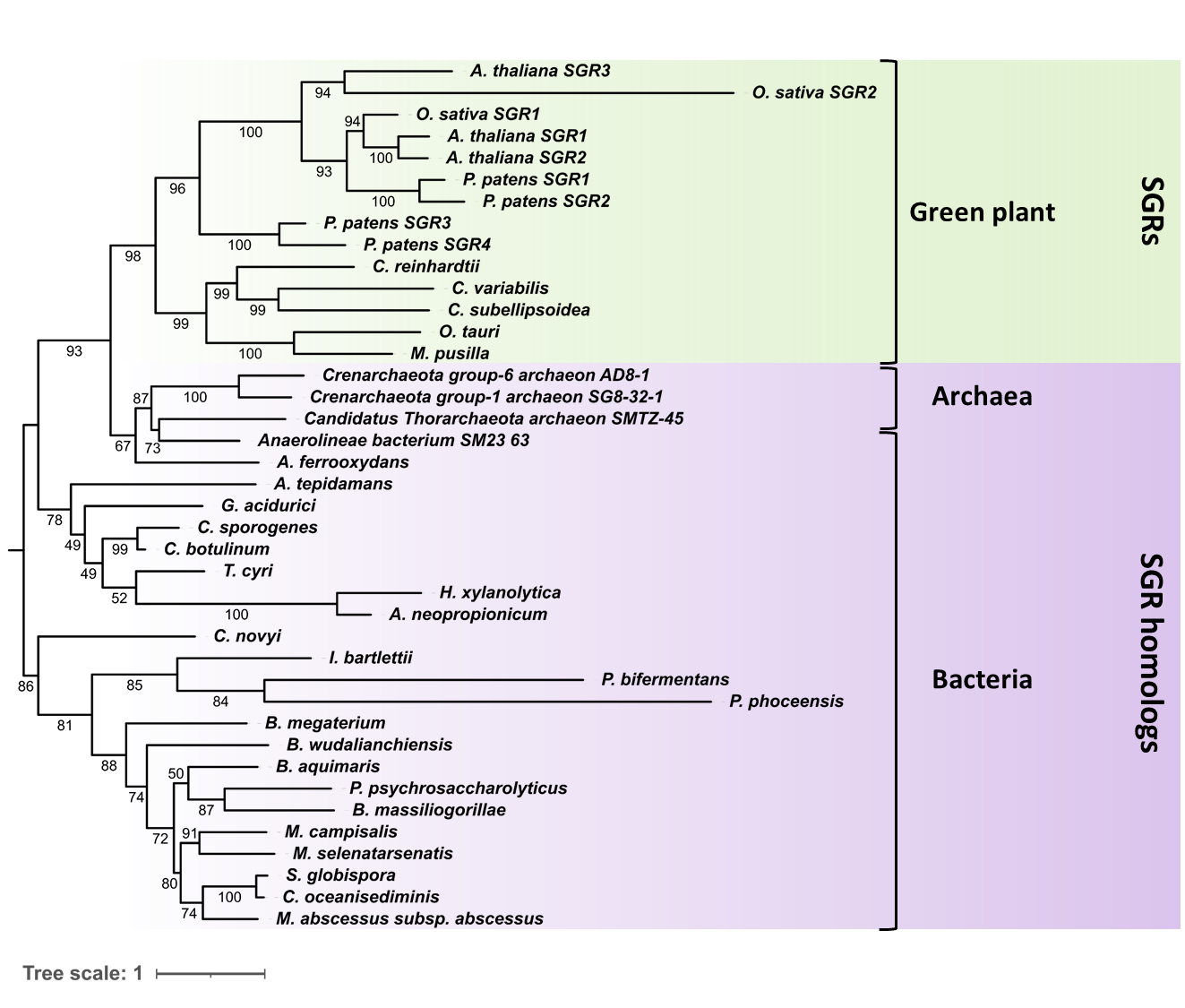


**Supplementary Figure 1:** Maximum likelihood phylogeny of protein sequences of SGRs and its homologs. Bootstrap values, based on 1000 replicates, have been shown on branch nodes.


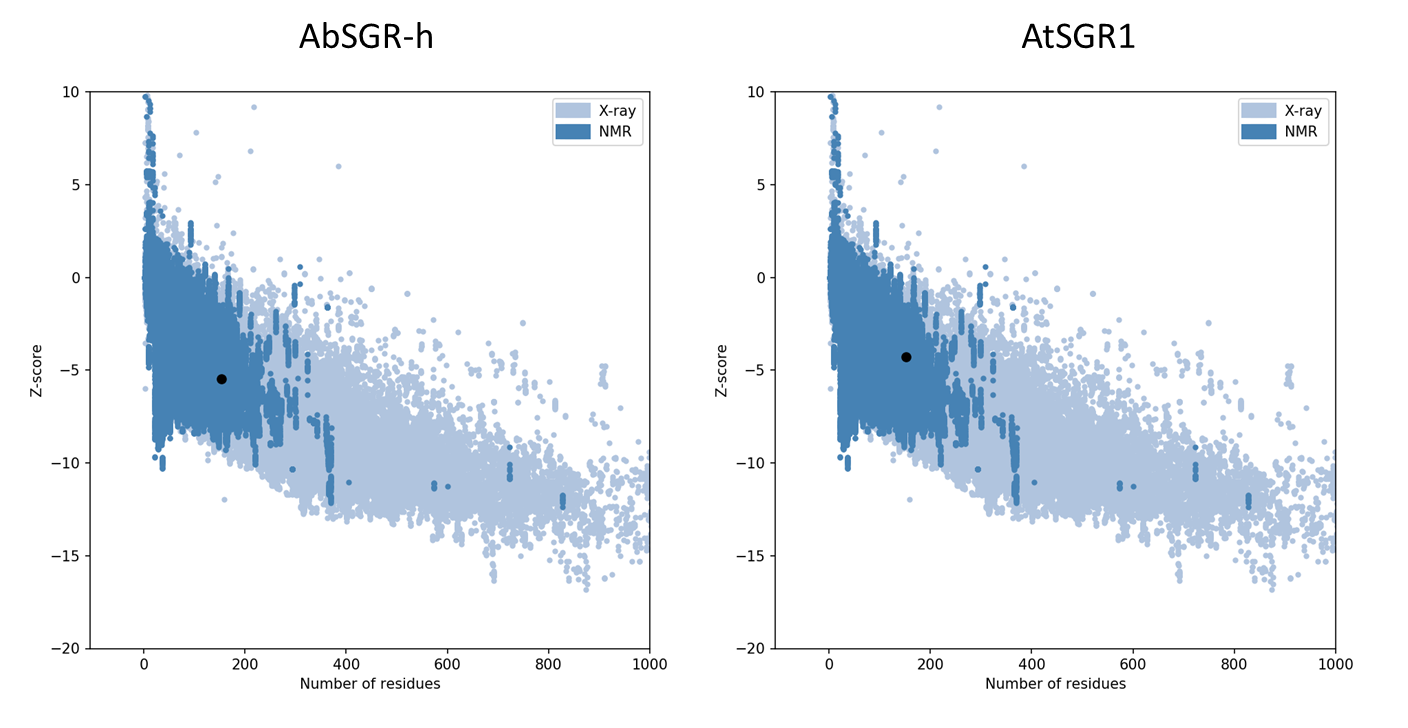


**Supplementary Figure 2:** ProSA plot of the two proteins showing Z-score. The regions with blue and light blue colors are about the groups of similar structures of protein, determined from NMR and X-Ray methods, respectively. The black dot in the plots represent the predicted SGR models.


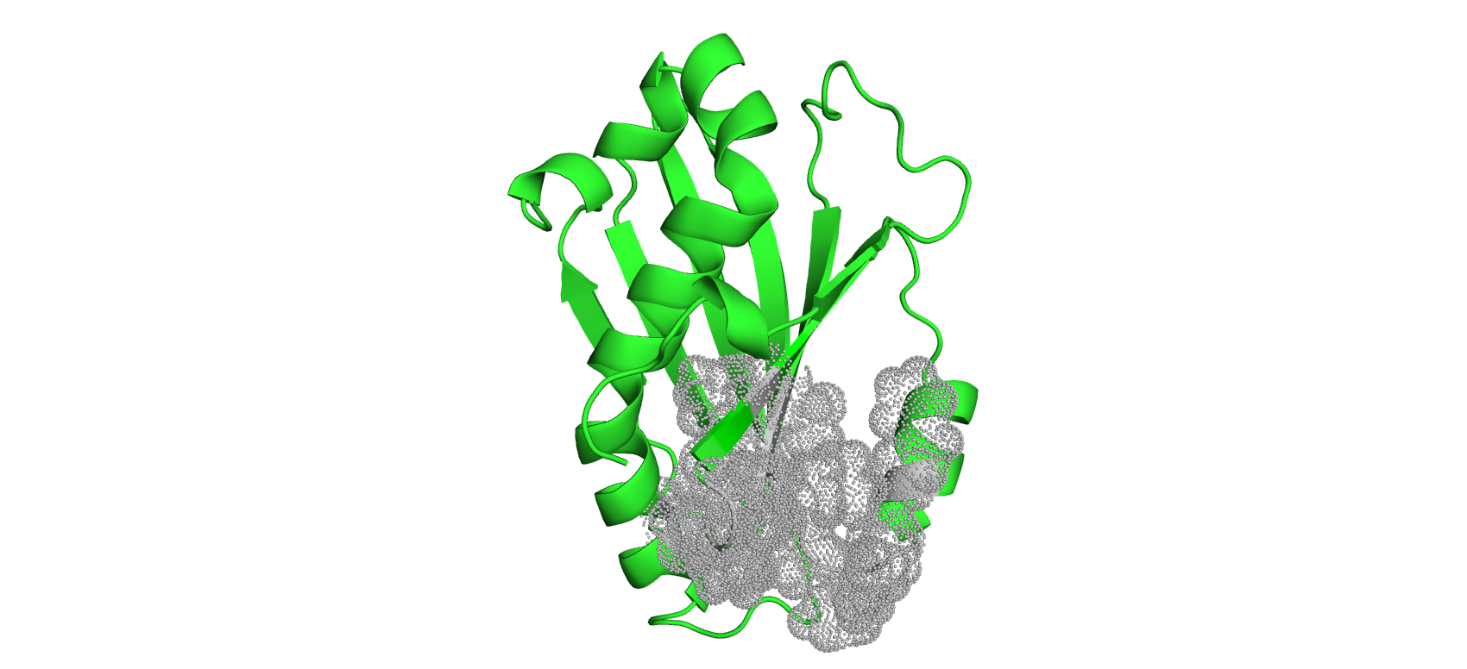


**Supplementary Figure 3:** Predicted binding cavity of AbSGR-h, as detected by the CavityPlus tool. The residues constituting the predicted binding site have been shown in dot representation.


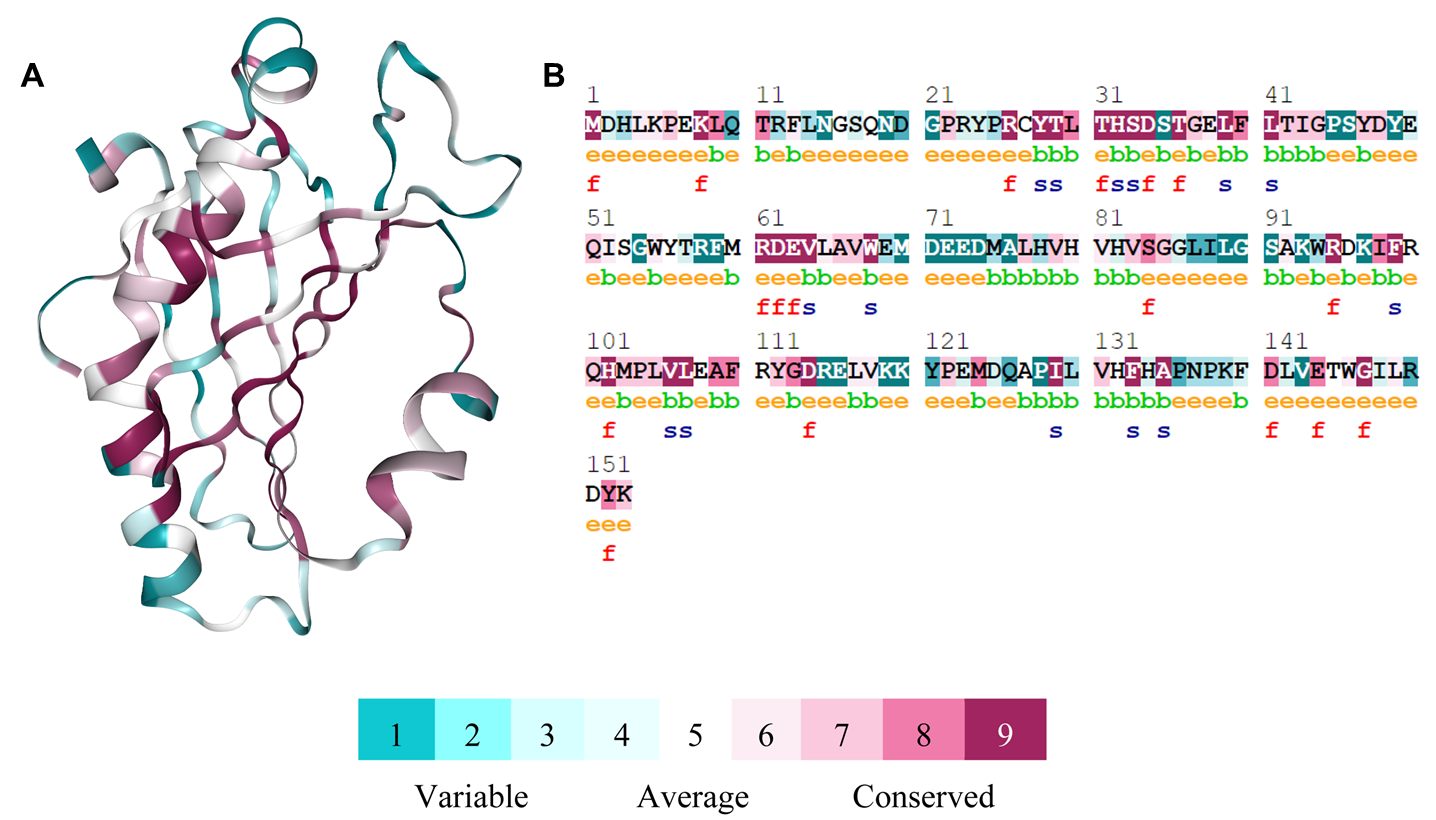


**Supplementary Figure 4:** Evolutionary conservation of amino acid residues in the three-dimensional structure of AbSGR-h protein by ConSurf analysis (A) and in the primary sequence of AbSGR-h by ConSeq analysis (B). ‘e’ refers to an exposed residue according to the neural-network algorithm; ‘b’ refers to a buried residue according to the neural-network algorithm; ‘f’ refers to a predicted functional residue (highly conserved and exposed); ‘s’ refers to a predicted structural residue (highly conserved and buried).


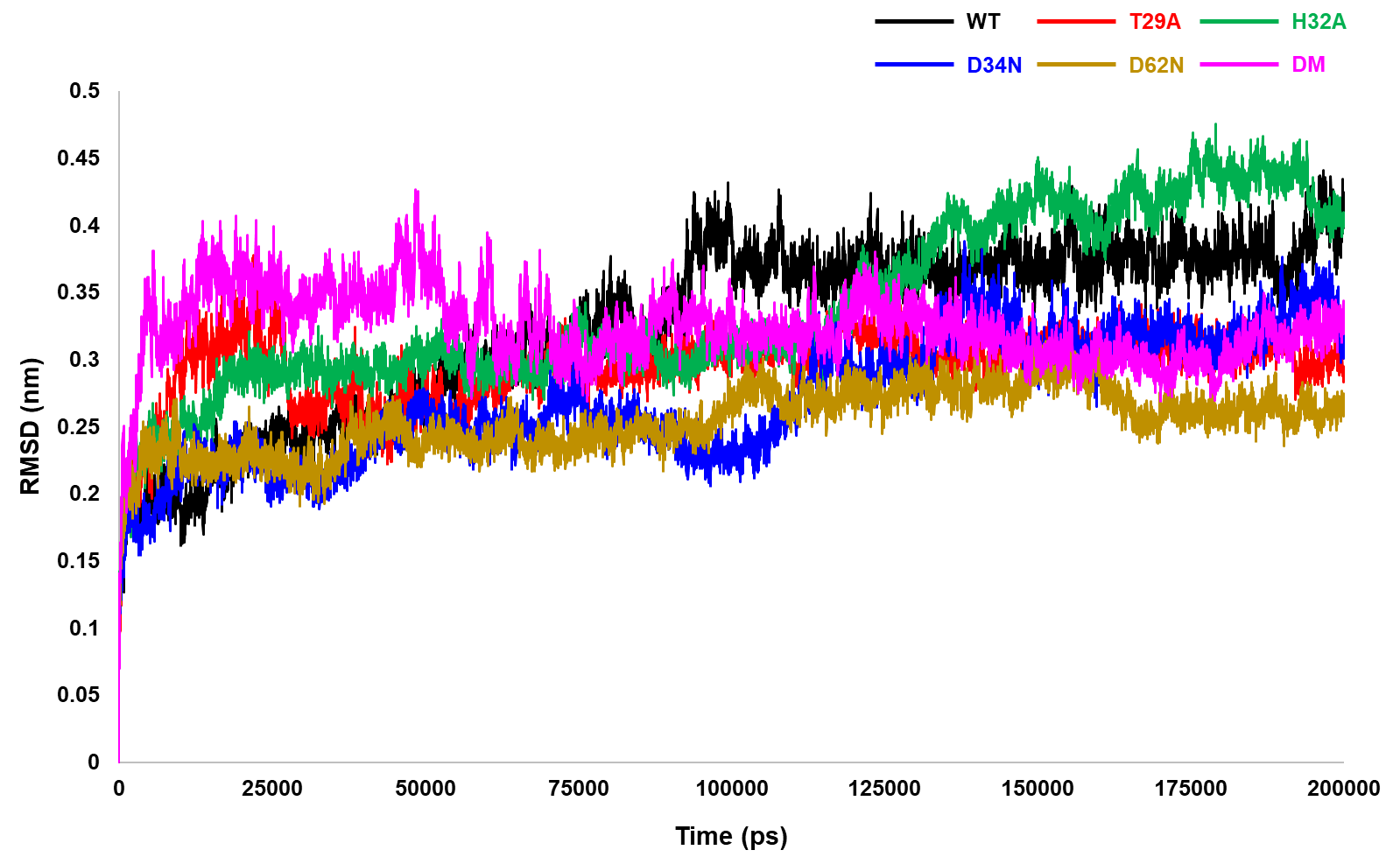


**Supplementary Figure 5:** RMSD plot of the backbone atoms of wild-type and mutant AbSGR-h proteins over the 200 ns trajectory of the MD production run.


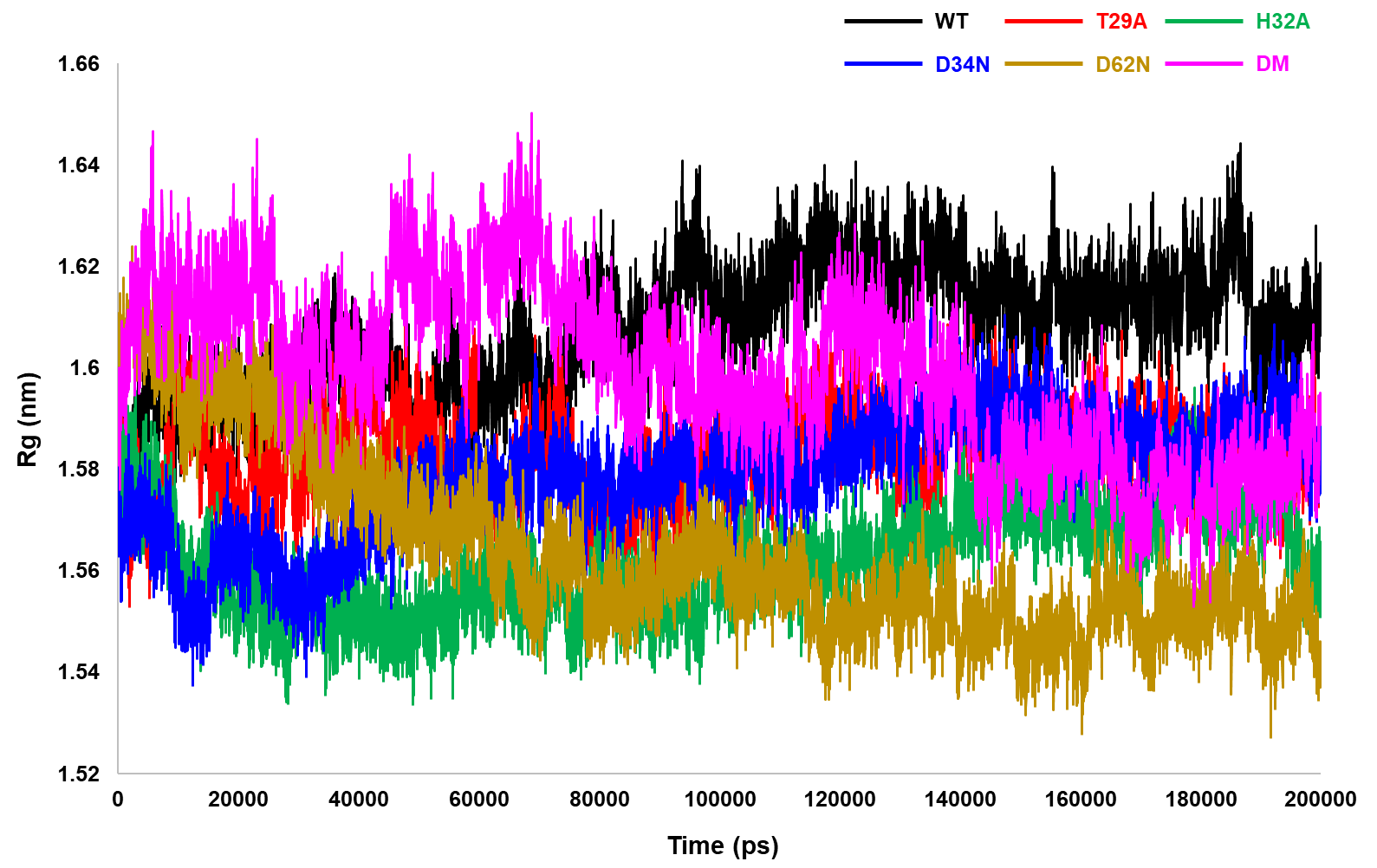


**Supplementary Figure 6:** Radius of gyration of wild-type and mutant AbSGR-h proteins over the 200 ns trajectory of the MD production run.
